# A key role for *S-*nitrosylation in immune regulation and development in the liverwort *Marchantia polymorpha*

**DOI:** 10.1093/jxb/erag171

**Published:** 2026-04-15

**Authors:** Nadra Tabassum, Justin Goodrich, Gary J Loake

**Affiliations:** Institute of Molecular Plant Sciences, School of Biological Sciences, Edinburgh University, Edinburgh EH9 3BF, UK; Department of Botany, University of Dhaka, Dhaka 1000, Bangladesh; Institute of Molecular Plant Sciences, School of Biological Sciences, Edinburgh University, Edinburgh EH9 3BF, UK; Institute of Molecular Plant Sciences, School of Biological Sciences, Edinburgh University, Edinburgh EH9 3BF, UK; University of Fribourg, Germany

**Keywords:** EvoMPMI, GSNOR, *Marchantia polymorpha*, nitric oxide, plant immunity, reactive nitrogen species, redox, *S*-nitrosylation

## Abstract

Nitric oxide (NO) is an important signalling molecule in flowering plant immunity. It rapidly accumulates in response to pathogen perception. In addition to its direct response to microbes, NO controls a range of defence responses primarily through *S*-nitrosylation. This process is a redox-dependent modification where a NO group attaches to the thiol of a cysteine residue, creating an *S*-nitrosothiol (SNO). To explore the role of *S*-nitrosylation more broadly, we characterized the single-copy *S*-*nitrosoglutathione reductase 1* (Mp*GSNOR1*) gene in the liverwort *Marchantia polymorpha*, a representative of a lineage widely divergent from flowering plants. We generated loss-of-function alleles using CRISPR/Cas9 genome editing. Disrupting Mp*GSNOR1* resulted in pronounced morphological alterations, highlighting the role of GSNOR1 in the structural development of *Marchantia*. Additionally, we show that Mp*GSNOR1* is essential for SNO homeostasis and immune function. Our results suggest that GSNOR was part of the tool kit of the ancestral land plant and functioned in immunity and development.

## Teaser

First evidence from a liverwort shows *S*-nitrosoglutathione reductase controls immunity and development via *S*-nitrosylation, revealing these regulatory roles as ancient traits of land plants.

## Introduction

The transition of plants from water to land about 470–515 million years ago significantly influenced the evolution of global biodiversity (Bowman *et al*., 2016, 2022; Romani *et al*., 2024). This transition is thought to have driven the evolution of a highly efficient and specialized plant immune system to protect against potential infection from co-evolving microbial pathogens (Carella *et al*., 2019; Poveda, 2020). Successful plant migration from water to land probably relied on the capacity of these plants to deal with various microorganisms that had already become successfully adapted to terrestrial conditions (Matsui *et al*., 2019; Yotsui *et al*., 2023).

Evolutionary molecular plant–microbe interactions is a rapidly emerging field that has contributed to advancing our knowledge regarding the emergence of early plant immune systems (Gimenez-Ibáñez *et al*., 2019; Redkar *et al*., 2022). In this regard, *Marchantia polymorpha* (hereafter *Marchantia*) is showing promise as an excellent model species for comparative studies to characterize evolution of traits and ancestral functions (Bowman *et al*., 2017, 2022; Alcaraz *et al*., 2018).

*Marchantia* is named after the French botanist Nicolas Marchant and has been studied for its life cycle and medicinal properties since the 16th century (Bowman *et al*., 2016). Interest in *Marchantia* as a model species has been rekindled both by technical advances such as the availability of a genome sequence and facile gene disruption via genome editing and by its phylogenetic position as an extant representative of a lineage that diverged from that of flowering plants at least 450 million years ago. It is readily transformed and has a relatively small genome of 250 Mb with low redundancy for many of its developmental and regulatory genes (Bowman *et al*., 2017; Montgomery *et al*., 2020; Iwasaki *et al*., 2021). *Marchantia* features a dominant haploid gametophyte generation in its life cycle, facilitating gene discovery and characterization through forward and reverse genetic screens (Shimamura, 2016). It reproduces both asexually, via clonal propagules termed gemmae, and sexually via different male (antheridiophore) and female (archegoniophore) structures borne on separate male and female individuals (Bowman *et al*., 2016; Shimamura, 2016). It has nine chromosomes, including a sex specific U or V chromosome that determines female or male identity, respectively (Shimamura, 2016).

Recent research has demonstrated that several phytopathogens, including *Xylaria cubensis*, *Colletotrichum* species, *Phytophthora palmivora*, *Pseudomonas syringae*, *Fusarium oxysporum* (Fo), and several fungal endophytes can grow well within and thrive in *Marchantia*. Visibly apparent symptoms of infections by these pathogens generally comprise disease symptoms like necrosis and chlorosis on the thallus surface (Carella *et al*., 2018; Redkar *et al*., 2022). At the molecular level, Grenz *et al*. (2025) demonstrated functional compatibility between *Pseudomonas syringae* effector complexes and conserved immune components in *Marchantia*, providing evidence for the evolutionary conservation of host–pathogen signalling mechanisms. In parallel, Robinson *et al*. (2025) reported naturally occurring interactions between *Pseudomonas* species and *Marchantia*, highlighting the ecological relevance of this liverwort–bacterium association. Beyond pathogenic interactions, recent studies have also revealed diverse outcomes of *Marchantia* associations with beneficial or opportunistic microbes. For example, a study of the interaction of *Marchantia* and various *Trichoderma* species has revealed a range of results, including enhanced resistance, growth promotion, and increased disease susceptibility (Poveda *et al*., 2023). The study also investigated patterns of tissue colonization and found that this additionally involves transcriptional activation of certain genes related to the defence system, forming such products as salicylic acid (SA) (Poveda *et al*., 2023). The expression of these functional responses depends on a conserved immune repertoire. This includes LysM-domain pattern-recognition receptors (MpLYK1 and MpLYR), nucleotide binding leucine-rich repeat (NLR)-like resistance factors, and parts of the SA pathway, which suggests that important elements of the land plant immune system likely existed in early diverging lineages (Matsui *et al*., 2019; Yotsui *et al*., 2023).

However, the immune structure of this liverwort is still mostly unexplored (Bowman *et al*., 2022). Recent research has highlighted the role of the thallus of *Marchantia* as a mediator of susceptibility (Carella *et al*., 2018; Iwakawa *et al*., 2021). In particular, the air chambers, which help with gas exchange, may serve as entry points for pathogens. *Marchantia* Mp*nop1* mutants, which do not develop these chambers, show greater resistance to *P. palmivora*, *Pseudomonas syringae* pv *tomato* (*Pst*), and *Agrobacterium tumefaciens*, indicating that a lack of access to internal tissues hinders colonization (Tsuboyama-Tanaka and Kodama, 2015; Carella *et al*., 2018; Iwakawa *et al*., 2021).

Interrogation of the *Marchantia* genome has uncovered 23 resistance (R)-like NLR genes (Bowman *et al*., 2017), which in angiosperms encode proteins that predominantly recognize the activity of pathogen effector proteins (Lin *et al*., 2012; Bowman *et al*., 2017). In *P. palmivora* infections, *in planta* expression of virulence genes, including RxLR effectors, proteases, and cell wall-degrading enzymes, has been observed, along with the formation of haustoria, highlighting pathogen strategies for immune evasion and host manipulation (Carella *et al*., 2018). In the case of *Pst*, mutants deficient in type III secretion (*hrcC–)* display significantly reduced virulence, while effectors like avrPto and avrPtoB suppress host immunity, mirroring their roles in flowering plants (Gimenez-Ibáñez *et al*., 2019).

In addition, *Marchantia* also produces the key immune signalling molecule SA in response to attempted pathogen infection. Further, the genome of *Marchantia* possesses homologues of all critical components associated with SA biosynthesis and signalling found in vascular plants (Lin *et al*., 2012; Gimenez-Ibáñez *et al*., 2019). By contrast, algal genomes only include select components of the SA molecular machinery, indicating that this system may have evolved during plant colonization of the land (Bowman *et al*., 2017; Gimenez-Ibáñez *et al*., 2019).

Moreover, recent findings on *Marchantia* Respiratory Burst Oxidase Homolog 1 (MpRBOH1), a key component in ROS production, suggest evolutionary conservation of ROS pathways across plant species (Chu *et al*., 2023). The dysregulation of nitric oxide (NO) homeostasis in *Marchantia S*-*nitrosoglutathione reductase 1* (Mp*GSNOR1*) mutants might disrupt the balance between NO and ROS pathways, impacting the overall plant immune response. This highlights how an imbalance in one signalling molecule, like NO, can influence the effectiveness of overall plant defence mechanisms.

In vascular plants, a key feature of the immune response following recognition of potential pathogens is the engagement of a rapid nitrosative burst, leading to the accumulation of NO and numerous other reactive nitrogen intermediates (Yu *et al*., 2014). These small redox-active molecules regulate a plethora of key immune-related functions, including the oxidative burst (Yun *et al*., 2011), SA-based signalling (Loake and Grant, 2007; Tada *et al*., 2008; Moreau *et al*., 2010), and immune-related transcriptional reprogramming (Cui *et al*., 2018; Imran *et al*., 2018). This regulation, through the transfer of NO bioactivity, predominantly occurs by *S*-nitrosylation, the reversible attachment of an NO moiety to a rare, highly reactive protein cysteine (Cys) thiol (SH), to form an *S*-nitrosothiol (SNO) (Xia *et al*., 2014; Yu *et al*., 2014; Stomberski *et al*., 2019). Thus, this redox-based, post-translational modification is akin to more well-established post-translational modifications (PTMs), such as phosphorylation (Sefton and Shenolikar, 2001; Cohen, 2002), in the control of protein function.

The extent of cellular *S*-nitrosylation can be controlled directly by Thioredoxin h5 (Trxh5) through the SNO reductase activity of this enzyme (Kneeshaw and Spoel, 2018) and indirectly, via the turnover of *S*-nitrosoglutathione (GSNO), which functions as a storage reservoir for NO bioactivity (Yun *et al*., 2016; Kneeshaw and Spoel, 2018), by GSNO reductase (GSNOR) (Feechan *et al*., 2005; Lee *et al*., 2008; Chen *et al*., 2009). This enzyme is required for resistance gene-mediated protection and systemic acquired resistance (Feechan *et al*., 2005; Wang, 2006; Tada *et al*., 2008; Wang *et al*., 2013). Furthermore, the absence of GSNOR1 inhibited the accumulation of SA and resulted in delayed and reduced expression of SA-dependent genes in Arabidopsis (Feechan *et al*., 2005). GSNOR1 function is therefore required for both SA biosynthesis and associated signal transmission (Feechan *et al*., 2005; Wang, 2006; Tada *et al*., 2008; Wang *et al*., 2013), altering their functions in pathogen defence (Tada *et al*., 2008; Yun *et al*., 2011).

Thus, while the pathogen-triggered nitrosative burst and cognate redox signalling is a key feature of the immune response in flowering plants, a potential role for this molecular machinery in the immune systems of other land plant lineages remains to be established. To address this deficiency, we identified an orthologue for *GSNOR* in *Marchantia* and subsequently generated a series of loss-of-function alleles for this gene by CRISPR/Cas9-based gene editing.

Similar to the phenotypes observed in vascular plant *GSNOR* mutants (Lee *et al*., 2008; Kwon *et al*., 2012), disruption of Mp*GSNOR* affected not only pathogen defence but also fundamental developmental processes. Mutants displayed reduced thallus growth, defective gemma cup and rhizoid formation, delayed sporophyte development, and increased susceptibility to *Pst* DC3000 infection. Recent work on MpRBOH1 further highlights the central role of ROS-generating enzymes in immunity and demonstrates that ROS-related signalling components are evolutionarily conserved across plant lineages (Chu *et al*., 2023). In this context, the altered NO homeostasis in Mp*GSNOR* mutants may disrupt the delicate balance between NO and ROS pathways, ultimately compromising the plant immune response.

These findings suggest that redox homeostasis, particularly through GSNOR-mediated NO signalling, involved in redox homeostasis during development and immunity, is conserved between the widely diverged lineages of liverworts and flowering plants. This implies the mechanism was likely present in the most recent common ancestor of land plants as part of its ancestral signalling network.

## Materials and methods

### Plant material and growth condition

The male accession Takaragaike-1 (Tak-1) of *Marchantia* was used as the wild type (WT) in this study. Gemmae of *Marchantia* were cultivated *in vitro* on half-strength Gamborg’s B5 medium supplemented with 1% agar (solid medium) or without agar (liquid medium). Cultures were maintained under a 16 h light/8 h dark photoperiod, with a light intensity of 110 µmol m^−2^ s^−1^, at 20 °C and a relative humidity of 60–65%. For bacterial infection experiments, 2- to 4-week-old plants were transferred to controlled environmental chambers maintained at 22 °C (range, 16–24 °C), 45–65% relative humidity, and short-day conditions (8 h of light; 110 µmol m⁻² s⁻¹).

### Phylogenetic analysis

The Arabidopsis GSNOR1 protein sequence was used in BLASTP searches to retrieve similar proteins from various databases as follows: Marpolbase (https://marchantia.info) for *Marchantia polymorpha*, *Physcomitrella patens*, *Selaginella moellendorffii*, *Azolla filiculoides*, *Amborella trichopoda*, *Spirogloea muscicola*, and *Chlamydomonas reinhardtii*; and Genbank (https://blast.ncbi.nlm.nih.gov/Blast.cgi) for *Cyanidioschyzon merolae* and *Picea sitchensis.* The *Cyanophora paradoxica* sequence was retrieved from the transcriptome as the Cyanophora genome project (https://data.jgi.doe.gov/).

Sequences with E-values less than 10^−42^ were selected and aligned using MAFFT implemented within the Geneious package (https://www.geneious.com) using the AUTO algorithm and BLOSUM62 scoring matrix. The alignment was manually trimmed, and positions with >30% gaps were stripped using the mask alignment tool in Geneious. The model selection tool within IQTree (https://iqtree.github.io/) was first used to select the LG+R5 model and the maximum likelihood tree inferred with IQ-TREE. The output tree was exported as a .svg file and edited using FigTree (http://tree.bio.ed.ac.uk/software/figtree/) and Inkscape (https://inkscape.org/).

### Generation of CRISPR/Cas9-mediated Mp*gsnor1* knockout mutants

Mp*gsnor1* knockout mutants in *Marchantia* were generated using CRISPR/Cas9 technology through *Agrobacterium*-mediated gemma (Agar Trap) transformation of Tak-1 (WT) gemmae (Tsuboyama and Kodama, 2014; Tsuboyama *et al*., 2018). Two guide RNAs (MpGNR_sgRNA1 and MpGNR_sgRNA2; Supplementary File Supplementary Table S1) targeting the second exon of Mp*GSNOR1* were designed using CRISPRdirect (https://crispr.dbcls.jp) and cloned into the pGE_En03 entry vector as per Sugano *et al*. (2018). The constructs were recombined into the Cas9-containing binary vector pMpGE011 and introduced into *A. tumefaciens* GV3101 (mp90). Two independent mutant alleles, Mp*gsnor1-1^ge^* and Mp*gsnor1-^4ge^*, were recovered. We maintained the primary transformants (G_1_ generation) as stable lines, and gemmae produced by these G_1_ plants (G_2_ generation) were collected and used for all subsequent phenotypic and molecular examinations.

Transformed lines were screened by PCR using gene-specific primers that amplified ∼459 bp fragments spanning the target site (primer sequences are listed in Supplementary Table S1). Agarose gel electrophoresis was used to detect larger insertions or deletions (indels). However, since many CRISPR-induced mutations were small indels undetectable by gel, a subset of transformants was randomly selected for Sanger sequencing to confirm the presence of mutations, particularly frameshifts likely to disrupt gene function. Sequence alignments against the WT Mp*GSNOR1* sequence were used to verify edits. Although the Cas9 transgene was not eliminated, periodic sequencing was used to verify the stability of the mutations across generations.

### Generation of complementation constructs

For complementation experiments in *Marchantia*, constructs expressing Arabidopsis (At)*GSNOR1* were generated using Gateway cloning technology. The At*GSNOR1* coding sequence was amplified from Arabidopsis cDNA using primers listed in Supplementary Table S1, cloned into the pDONR™221 vector (Thermo Fisher Scientific) by BP clonase-mediated recombination according to the manufacturer’s instructions, and verified by sequencing. Following recombination by Gateway LR Clonase II enzyme (Thermo Fisher Scientific), the At*GSNOR1* sequences were inserted downstream of a *35S* promoter in the Gateway-compatible binary vector pMpGWB102. Constructs were introduced into *A. tumefaciens* strain GV3101 (mp90) by heat shock transformation and subsequently used to transform the Mp*gsnor1-1^ge^* and Mp*gsnor1-4^ge^* mutant backgrounds by G-Agar trap (gemma transformation) methods (Tsuboyama *et al*., 2018). The presence of the T-DNA construct in the putative transformants were confirmed by PCR using At*GSNOR1-*specific primers (Supplementary Table S1). The complementation line *pro35S:*At*GSNOR1^ge^* in Mp*gsnor1-4^ge^* background was subsequently used for the experiments.

### Transformation of *Marchantia*

Transformation of *Marchantia* was performed using the G-agar trap method (Tsuboyama and Kodama, 2018). Transformants were selected on half-strength Gamborg’s B5 medium containing 1% agar, 100 µg ml^−1^ cefotaxime, and 0.5 µM chlorsulfuron to isolate non-chimeric transgenic gemmae. For complementation of the Mp*gsnor1* mutants, selection was performed on half-strength Gamborg’s B5 medium containing 1% agar, 100 µg ml^−1^ cefotaxime, and 10 µg ml^−1^ hygromycin to recover super transformants (carrying both the Cas9 gene editing T-DNA and the At*GSNOR1* construct T DNA).

### Bacterial strains and inoculation methods

*Pseudomonas syringae* pv. *tomato* DC3000 (*Pst* DC3000) was cultivated on Luria–Bertani (LB) medium supplemented with rifampicin (50 µg ml^−1^) at 28 °C. Overnight cultures were grown at 28 °C with shaking at 300 rpm. Inoculation methods followed the procedure described by Gimenez-Ibáñez *et al*. (2019). Briefly, *Marchantia* gemmalings were grown on Whatman filter paper over half-strength Gamborg’s B5 agar medium at 21 °C under a 16 h light (110 µmol m⁻² s⁻¹)/8 h dark cycle. Plants were inoculated by dipping in a bacterial suspension of 10^8^ CFU ml^−1^ (OD_600_=0.2) containing 0.04% Silwet L-77 for 5 min. Post-inoculation, plants were transferred to soil under short-day conditions. Bacterial growth was quantified by plating serial dilutions of thallus disc homogenates harvested 3 d or 5 d post-inoculation.

### *S*-nitrosoglutathione reductase activity assay

GSNOR activity was determined by monitoring NADH consumption at 340 nm, following established methods (Feechan *et al*., 2005). Protein extracts (100 µg) from WT and mutant plants were incubated at 25 °C in a 1 ml reaction mixture containing 20 mM Tris–HCl (pH 8.0), 0.2 mM NADH, and 0.5 mM EDTA. Reactions were initiated by adding GSNO at a final concentration of 300 µM. Activity was expressed as nmol NADH consumed per min per mg of protein. GSNOR1 activity was measured in three independent experiments using five biological replicates in each experiment.

### Biotin-switch assay for total *S*-nitrosylation measurement

The assay was performed essentially as described by Jaffrey and Snyder (2001) with modifications for *Marchantia*. Four-week-old *Marchantia* plants were inoculated with *Pst* DC3000 or mock-treated with 10 mM MgCl_2_. Approximately 200 mg of thallus tissue (fresh or stored at −80 °C) was ground to a fine powder in liquid nitrogen and homogenized with four volumes of extraction buffer (250 mM HEPES, 1 mM EDTA, 0.1 mM neocuproine, pH 7.7, supplemented with 0.5% (v/v) Triton X-100 and 1× protease inhibitor cocktail). Homogenates were divided into 1.5–2 ml microcentrifuge tubes and centrifuged at ∼20 000× *g* for 15 min at 4 °C. The supernatant was filtered through a 0.45 µm syringe filter, and protein concentration was determined by absorbance at 280 nm (NanoDrop). Samples were normalized to 200 µg protein in HEN buffer (250 mM HEPES, 1 mM EDTA, 0.1 mM neocuproine, pH 7.7) and aliquoted into 100 µl volumes. For free thiol blocking, 300 µl blocking buffer (HEN containing 5% SDS and 50 mM *N*-ethylmaleimide (NEM) was added to each sample. Tubes were wrapped in aluminium foil and incubated at 50 °C for 20 min with intermittent vortexing. Proteins were precipitated by adding two volumes of ice-cold 100% acetone and incubating at −20 °C for at least 20 min (or overnight). Pellets were collected by centrifugation (∼20 000× *g*, 5 min, 4 °C), washed three times with 70% (v/v) acetone at 4 °C, and air-dried briefly in the dark. Pellets were resuspended in 96 µl HENS buffer (HEN containing 1% SDS), and freshly prepared sodium ascorbate (500 mM, 12 µl) and biotin-HPDP (12 µl, final 0.4 mM; Thermo Fisher Scientific) were added. Reactions were incubated at room temperature for 1–2 h on a rocker, protected from light. Control reactions were done without sodium ascorbate. After labelling, 120 µl 2× SDS loading buffer was added, and samples were loaded onto SDS–PAGE gels without boiling. Following electrophoresis and transfer to polyvinylidene difluoride membranes, *S*-nitrosylated proteins were detected using horseradish peroxidase-conjugated anti-biotin antibody (Cell Signaling Technology, cat. no. 7075) and visualized by chemiluminescence. The biotin switch assay was performed in three independent experiments using biological replicates.

### Confocal imaging of pathogen-induced nitric oxide accumulation

Endogenous NO accumulation in *Marchantia* was analysed by confocal microscopy using the fluorescent probe diaminofluorescein-FM diacetate (DAF-FM DA). Two-week-old *Marchantia* thalli possessing rhizoids were incubated in MES buffer (10 mM MES, 50 mM KCl, pH 6.15) for 10 min, after which thin sections of the thallus with rhizoids were prepared and mounted on depression slides. Samples were incubated with DAF-FM DA (1 μl ml^−1^; stock 10 mg ml^−1^) for 10 min at room temperature in the dark, followed by washing with MES buffer to remove excess dye. NO-dependent fluorescence was imaged using a Leica SP8 confocal microscope. For pathogen-induced NO detection, *Marchantia* samples were inoculated with *Pst* DC3000 and imaged at the indicated time points using the same procedure. To confirm signal specificity, samples were treated with the NO scavenger 2-(4-carboxyphenyl)-4,4,5,5-tetramethylimidazoline-1-oxyl-3-oxide (cPTIO; 100 μM). Images were acquired using a ×40 objective (zoom 1.4, pinhole 1 AU) with Z-stacks collected at 1 μm intervals. Excitation was set at 480 nm and emission was detected at 515 nm. Fluorescence intensity was quantified using Leica LAS X and ImageJ software. All experiments were independently repeated three times, with each replicate consisting of five biological samples per genotype.

### RNA extraction and cDNA synthesis

Total RNA was extracted from approximately 100 mg of 3-week-old *Marchantia* thalli using TRIzol followed by purification with Qiagen spin columns according to the manufacturer’s protocol. RNA quality and concentration were assessed by agarose gel electrophoresis and NanoDrop spectrophotometry, respectively. cDNA synthesis was performed using either the High-Capacity cDNA Reverse Transcription Kit (Thermo Fisher Scientific) for isolating the Arabidopsis GSNOR1 cDNA or the LunaScript RT SuperMix Kit (NEB, cat. no. E3010) for qRT-PCR analyses following the guidelines of the manufacturer.

### Quantitative real-time PCR

Quantitative real-time PCR (qRT-PCR) reactions were performed using Luna Universal qPCR Master Mix (NEB, cat. no. M3003). Each 20 µl reaction included 10 µl master mix, 0.5 µl forward and reverse primers (10 µM each), and 1 µl cDNA template. Reactions were carried out in a Roche LightCycler 480 instrument under the following cycling conditions: initial denaturation at 95 °C for 60 s, followed by 45 cycles at 95 °C (15 s) and 60 °C (30 s). Melt curve analysis (60–95 °C) verified amplification specificity. Relative gene expression was calculated using the ΔΔ*C*_t_ method with Mp*ACT* (*Marchantia Actin*) as a reference gene (Saint-Marcoux *et al*., 2015). Three biological and three technical replicates were performed per treatment. Primer sequences are provided in Supplementary Table S1.

### Differential interference contrast microscopy

Morphological features of thalli and rhizoids were visualized using a Nikon Eclipse E600 microscope equipped with differential interference contrast (DIC) illumination and polarization filters. Samples were mounted on glass microscope slides and examined at magnifications of ×10, ×20, or ×40. Images were captured using an attached digital camera, and scale bars were added using the accompanying camera software for accurate size referencing.

### Statistical analysis

All statistical analyses were conducted utilizing GraphPad Prism (version 10.0; GraphPad Software, Inc., Boston, MA, USA). Phenotypic alterations, GSNOR activity, biotin switch assay outcomes, qRT-PCR expression data, and pathogenicity assessments were analysed employing an unpaired two-tailed Student’s *t*-test, as indicated in the figure legends and the corresponding number of replicates for each experiment. Results were considered statistically significant at *P*<0.05.

## Results

### *Marchantia* has a single *GSNOR1* orthologue

**Fig. 1.**
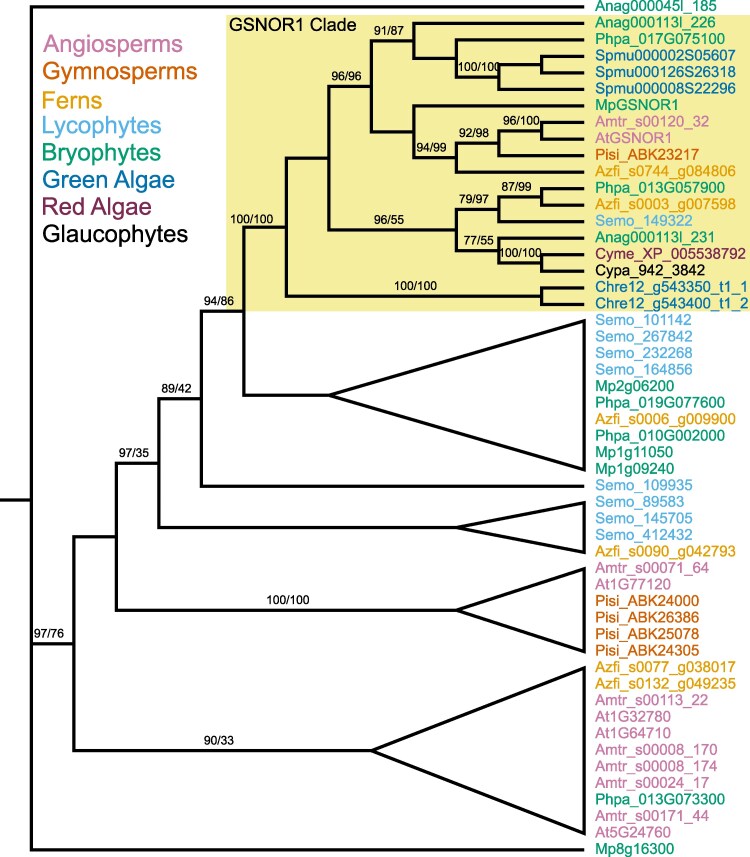
Phylogenetic tree of *S*-nitrosoglutathione reductase 1 (GSNOR1) and related proteins. Unrooted maximum likelihood tree created with IQTree and shown as a cladogram. Branches with ultrafast bootstrap and SH-aLRT branch support values exceeding 95% and 80% have high confidence. Ultrafast bootstrap values <75% are not shown. The strongly supported GSNOR1 clade is highlighted, other clades shown as cartoons. Species abbreviations: Amtr, *Amborella trichopodai*; Anag, *Anthoceros agrestis*; At, *Arabidopsis thaliana*; Azfi, *Azolla filiculoides*; Chre, *Chlamydomonas reinhardtii*; Cyme, *Cyanidioschyzon merolae*; Cypa, *Cyanophora paradoxa*; Mp, *Marchantia polymorpha*; Phpa, *Physcomitrella patens*; Psi, *Picea sitchensis*; Semo, *Selaginella moellendorffi*; Spmu, *Spirogloea muscicola*. The different taxonomic groupings are coloured as indicated top left.

GSNOR1 is a member of the class III alcohol dehydrogenases (ADHs) distinguished by its use of GSNO as substrate and NADH as cofactor. BLASTP searches using Arabidopsis At*GSNOR1* (At5g43940) as query against the *Marchantia* proteome retrieved the Mp1g16170 product (hereafter MpGSNOR1) as the best match. The two proteins showed very high similarity throughout their lengths (81% identity over 374 amino acids). Several other *Marchantia* proteins also had high similarity over the full length of At*GSNOR1*—for example, the Mp2g02600 and Mp8g16300 products had 53% and 51% identity, respectively. To explore the relationships further, we made a maximum likelihood phylogenetic analysis (Fig. 1) sampling GSNOR1 and other class III ADHs from diverse plant and algal lineages. This resolved a very strongly supported clade that contained GSNOR1 orthologues from all the major lineages of the *Archaeplastida* (plants, green algae, red algae, and glaucophytes) and confirmed that Mp*GSNOR1* is the only *Marchantia* orthologue—in other words, MpGSNOR1 is more closely related to GSNOR1 from Arabidopsis, algae, etc. than it is to Mp2g02600 or any other *Marchantia* protein. The other *Marchantia* proteins with similarity to GSNOR1 mapped elsewhere in the phylogeny, suggesting that they are ADH class enzymes but unlikely to use GSNO as substrate.

Consistent with this phylogenetic placement, multiple sequence alignments (Supplementary Fig. S1) revealed that while all *Marchantia* proteins conserved the residues coordinating the catalytic and structural zinc atoms, only MpGSNOR1 retained the residues essential for NAD(H) binding and GSNO recognition. In addition, MpGSNOR1 uniquely conserved several solvent-accessible cysteine residues previously implicated in redox-based PTMs of AtGSNOR1, whereas these cysteines were absent or poorly conserved in other *Marchantia* ADH-like proteins (Supplementary Fig. S1).

**Fig. 2.**
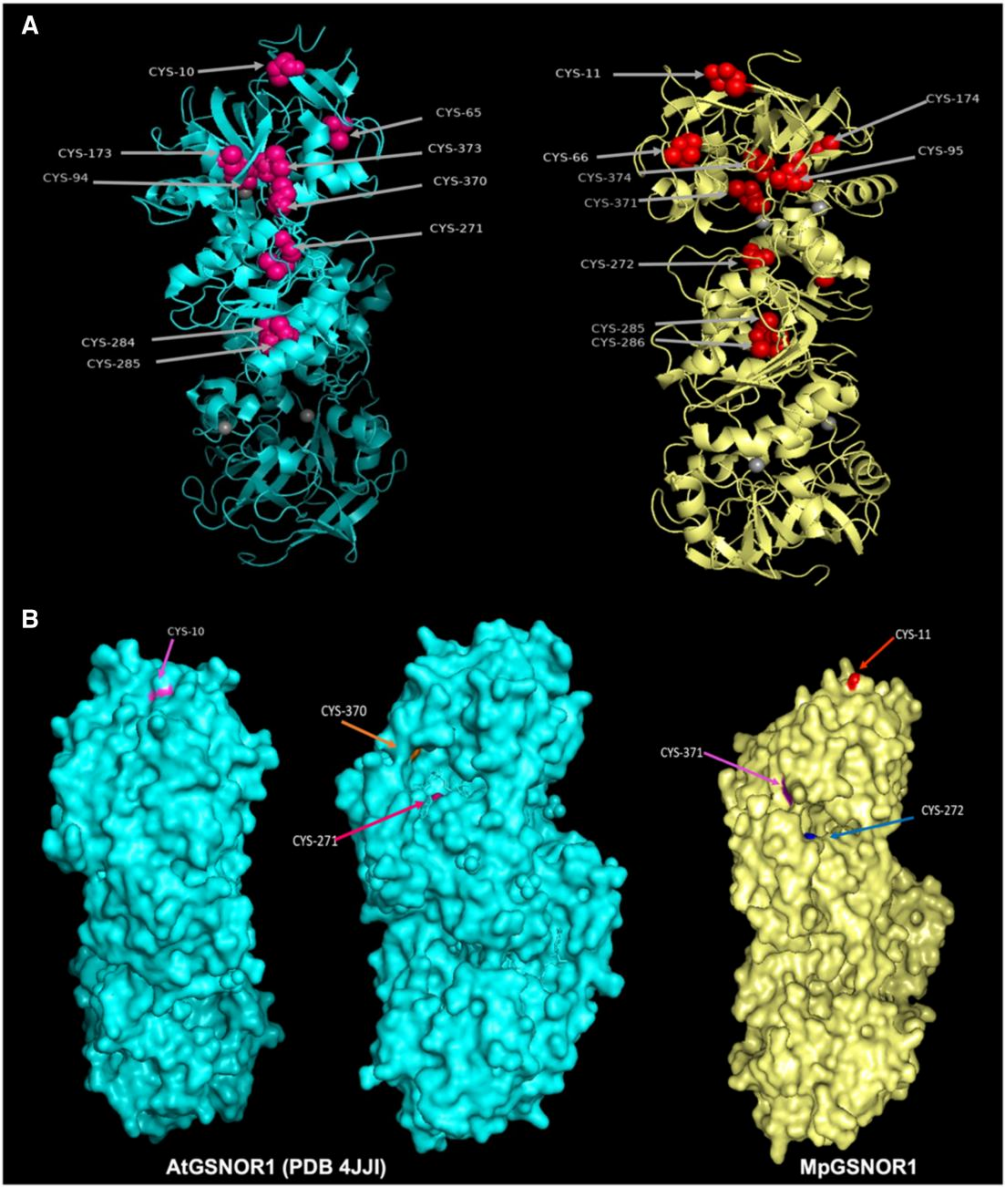
Conservation and solvent accessibility of cysteine residues in *S*-nitrosoglutathione reductase 1 (GSNOR1) from Arabidopsis and *Marchantia*. (A) Modelled structure of AtGSNOR1 (PDB 4JJI) (blue) and MpGSNOR1 (generated using SWISS-MODEL) (yellow), highlighting conserved active site cysteine residues. (B) Ex-zinc cysteines are annotated, and colour coding indicates specific Cys residues: Cys-10 (pink), Cys-271 (purple), and Cys-370 (orange) in AtGSNOR1; Cys-11 (red), Cys-272 (blue), and Cys-371 (violet) in MpGSNOR1. The protein models are oriented to emphasize solvent accessibility, illustrating the relative surface exposure and conservation of these critical residues across species. Images were generated in PyMOL.

Structural analysis using SWISS-MODEL and PyMOL showed that Mp*GSNOR1* also retains the non-zinc-coordinating cysteine residues found in At*GSNOR1* (Fig. 2). This finding further supports its evolutionary stability. The presence of these conserved structural elements, along with its classification in the medium-chain dehydrogenase/reductase (MDR) enzyme family, suggests that Mp*GSNOR1* functions similarly to At*GSNOR1* in maintaining cellular redox balance.

To determine whether MpGSNOR1 shares the same functional domain as AtGSNOR1, we analysed both proteins using the NCBI Conserved Domain Database (CDD). This revealed that MpGSNOR1 possesses an identical domain architecture to AtGSNOR1, including the characteristic *S*-(hydroxymethyl)glutathione dehydrogenase/Class III ADH domain (Domain Architecture ID 10169723) associated with the MDR family (Supplementary Fig. S2). The boundaries and composition of the conserved domains were nearly identical in both species, supporting the inference that MpGSNOR1 retains the canonical structural features required for GSNOR catalytic function.

Overall, these findings suggest that GSNOR1 was present in the last common ancestor of the land plants and has been conserved in *Marchantia* as a single copy gene. The strong similarity between GSNOR1 in Arabidopsis and *Marchantia*, along with their shared structural features and key functional residues, suggests potential biochemical and functional similarities.

### Generation of independent Mp*gsnor1* loss-of-function mutants by gene editing

To investigate the functional role of MpGSNOR1, we generated loss-of-function mutants using CRISPR/Cas9-mediated genome editing. Two guide RNA (sgRNA1 and 2) target sites were designed within the second exon of Mp*GSNOR1* (Mp1g16170) and selected for high specificity and minimal off-target effects (Supplementary Fig. S3A; Supplementary Table S1). Transgenic lines were generated through *Agrobacterium-*mediated transformation of WT (Tak1) gemmae, and multiple deletion alleles were identified: Mp*gsnor1-1^ge^* (1 bp deletion) and Mp*gsnor1-4^ge^* (4 bp deletion) were independently created using sgRNA1 and 2, respectively and were selected for further characterization. Sequence analysis of the Mp*gsnor1-1^ge^* and Mp*gsnor1-4^ge^* deletion alleles revealed frameshift mutations that introduce premature stop codons, thereby disrupting the MpGSNOR1 open reading frame and predicted to inactivate the MpGSNOR1 (Supplementary Fig. S3C). Translation of the mutant sequences showed that both deletions cause an immediate shift in the reading frame, resulting in altered amino acids near the N-terminus and the appearance of a premature stop codon shortly downstream. These truncations eliminate most of the conserved catalytic and cofactor-binding regions of the protein, strongly suggesting that the encoded proteins are non-functional.

### MpGSNOR1 regulates growth and development in *Marchantia*

**Fig. 3.**
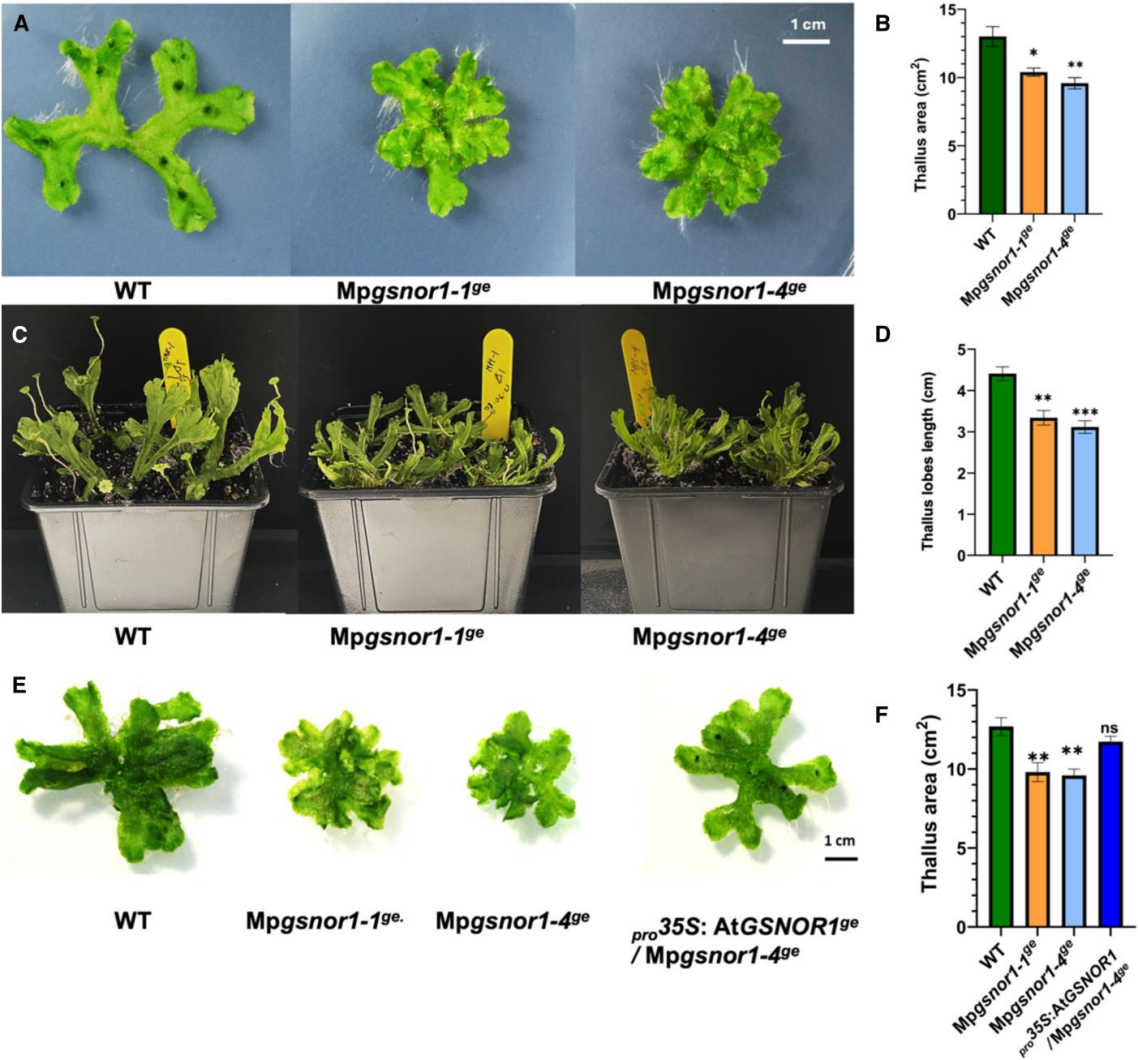
Thallus growth phenotypes of Mp*gsnor1* mutants and complementation line. (A) Representative images of 3- to 4-week-old wild-type (WT, Tak-1) and Mp*gsnor1* mutant lines (Mp*gsnor1-1^ge^* and Mp*gsnor1-4^ge^*) grown on half-strength Gamborg’s B5 medium from gemmae. (B) Quantification of thallus area (*n*=5). (C) Eight-week-old plants grown on sterilized soil. (D) Quantification of thallus area (cm^2^) and thallus lobe length (*n*=5). (E) Comparison of thallus growth among WT, Mp*gsnor1-1^ge^* and Mp*gsnor1-4^ge^* mutants, and the complemented line (*_pro_35S*: At*GSNOR1^ge^*/Mp*gsnor1-4^ge^*). Plants (4 weeks old) were grown on half-strength Gamborg’s B5 medium from gemmae. (F) Thallus area (cm^2^) quantification (*n*=5). Scale bars=1 cm for all panels. Statistical significance compared with WT is indicated in all plots (*P<0.05, **P<0.01, unpaired Student’s *t*-test), error bars represent SEM.

The Mp*gsnor1* loss-of-function mutations significantly impaired growth and development. When cultivated on Gamborg's B5 medium, both mutants exhibited a stunted phenotype characterized by reduced thallus size and altered growth morphology. Comparing Mp*gsnor1-1^ge^* and Mp*gsnor1-4^ge^* to the WT (Tak1), the thallus area decreased by 20.06% (*P*<0.05) and 26.98% (*P*<0.01), respectively (Fig. 3A, B). After 4 weeks, the WT thallus looked flatter and more dispersed, while the thallus lobes of the Mp*GSNOR1* mutant plants were densely arranged and twisted upward. A significant morphological divergence was also observed in soil-grown plants, with WT thallus lobes significantly (*P*<0.01) longer than those of the mutants (Fig. 3C, D). Complementation of the Mp*gsnor1-4^ge^* mutant with the Arabidopsis At*GSNOR1* gene (*_pro_35S*:At*GSNOR1*^ge^) in the Mp*gsnor1-4^ge^* background efficiently restored the thallus growth abnormalities observed in the mutant lines. Thallus area in the complemented lines approached 90% of WT levels (Fig. 3E, F). The growth morphology of complemented lines was also nearly identical to that of the WT, indicating that the Mp*GSNOR1* mutant phenotype was complemented.

### MpGSNOR1 promotes the growth of rhizoids

**Fig. 4.**
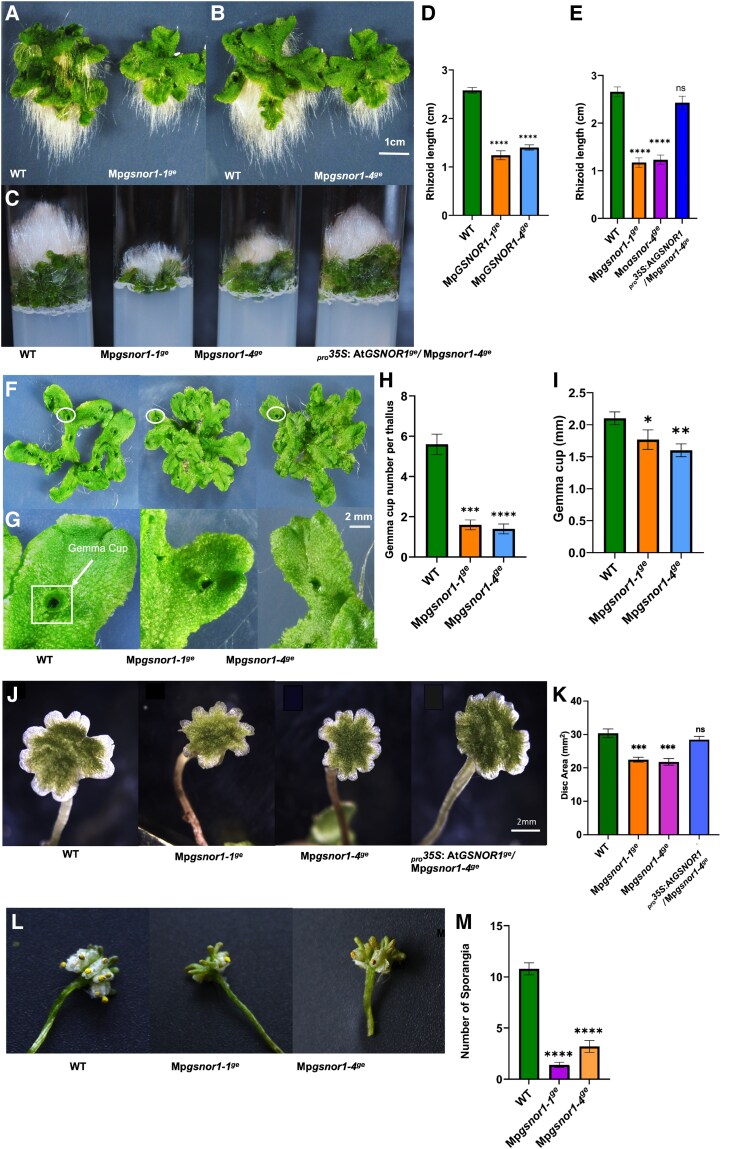
Phenotypic characterization of rhizoids, gemma cup, and reproductive structures of Mp*gsnor1* loss-of-function mutants and complementation lines. (A–E) Rhizoid development: representative images of different genotypes grown for 4 weeks on half-strength Gamborg’s B5 medium. (A, B) Plants grown on vertical plates showing rhizoid development. (C) Plants grown on inverted plates to visualize negatively phototrophic rhizoids growing away from the light and into the air. (D, E) Quantification of rhizoid length (*n*=5). (F–I) Gemma cup development: representative images and quantification of gemma cup number per thallus and gemma cup diameter in wild type (WT) and mutant lines. (H, I) Mean gemma cup number and diameter (mm) (*n*=5). (J) Antheridial disc development. (K) Quantification of antheridial disc area (*n*=5). (L, M) Representative images and quantification of sporophytes with closed sporangia in WT and mutant lines grown on half-strength Gamborg’s B5 medium (*n*=5). In all plots, error bars indicate SEM, significance relative to WT is indicated (ns, not significant; *P<0.05, **P<0.01, ***P<0.01, ****P<0.0001—based on an unpaired Student’s *t*-test). Scale bars=1 cm (A–C), 2 mm (H, I, K).

Rhizoids are long, tubular protrusions that emerge from epidermal cells on the abaxial surface of the thallus and primarily function in anchorage. There are two morphologically distinct types of rhizoids, smooth and pegged, with the latter specialized for water transport (Duckett *et al*., 2014; Lu *et al*., 2024). Notably, rhizoid development in bryophytes and root hair formation in angiosperms share a conserved genetic basis, with RSL class I basic helix–loop–helix transcription factors controlling the development of these specialized single-celled structures in the last common ancestor of land plants (Proust *et al*., 2016). Rhizoid length in Mp*gsnor1* mutants was significantly (*P*<0.001) reduced by 46–52% compared with WT (Fig. 4A–E). Like WT, mutant plants formed both smooth and pegged rhizoids (Supplementary Fig. S4). Importantly, in the complementation line *_pro_35S*:At*GSNOR1/*Mp*gsnor1-4^ge^*, rhizoid development was restored (Fig. 4C, E). Approximately 92% of the rhizoid length lost in the mutants was recovered, indicating that Arabidopsis *GSNOR1* can compensate for Mp*GSNOR1* deficiency. This finding demonstrates the functional equivalence of these genes, indicating that the observed defects in Mp*GSNOR1* mutants are specifically attributable to disruption of Mp*GSNOR1* rather than off-target effects. Furthermore, these results support the high degree of functional conservation of GSNOR1 across species.

### MpGSNOR1 loss can lead to reduced and delayed development of vegetative reproductive structures

Gemmae, formed within gemma cups on the dorsal thallus, enable clonal propagation in *Marchantia*. In Mp*GSNOR1* loss-of-function mutants, gemma cup formation was significantly impaired. Compared with WT, gemma cup production was reduced by 72% and 76%, respectively, after 4–5 weeks in growth medium (Fig. 4F–I). Mutant plants exhibited delayed gemma cup initiation, reduced gemma cup size, and slower maturation rates. Statistical analysis confirmed these differences are significant (*P*<0.001). These findings suggest that Mp*GSNOR1* plays a crucial role in regulating asexual reproductive development.

Complementation with *pro35S:*At*GSNOR1^ge^*/Mp*gsnor1-4^ge^* restored gemma formation, reaching 89% of WT levels (Supplementary Fig. S5). Gemma cups in the complemented line also recovered to approximately 93% of WT size, closely resembling normal development.

### MpGSNOR1 promotes the development of sexual reproductive organs

The development of sexual reproductive structures was significantly delayed in Mp*GSNOR1* mutants. Exposure to far-red enriched light (730 nm) induced antheridia formation in WT plants within 3–4 weeks of treatment, whereas Mp*gsnor1-1^ge^* and Mp*gsnor1-4^ge^* mutants exhibited a pronounced delay, with initiation occurring only after 6–7 weeks. Additionally, mutant plants developed smaller antheridial discs compared with WT. The average area of antheridial discs in Mp*gsnor1-1^ge^* and Mp*gsnor1-4^ge^* was distinctly smaller than WT with 26.1% and 28.4% reduction in mutant lines relative to WT (Fig. 4J, K).

Complementation with At*GSNOR1* restored reproductive defects. The complementation line showed a recovery of antheridia formation timing and antheridial disc size, reaching 90% of WT levels (Fig. 4L, M). Mp*GSNOR1* therefore promotes the initiation and development of reproductive structures, underscoring its role in controlling the switch from vegetative to reproductive growth.

Crosses between mutant (Mp*gsnor1-1^ge^* and Mp*gsnor1-4^ge^*) and WT (Tak-2) female plants revealed a reduction in sporophyte formation (Fig. 4L) with Mp*gsnor1-1^ge^* and Mp*gsnor1-4^ge^* siring only 87% and 70% of sporophytes compared with WT males, respectively (*P*<0.001). In some cases, sporophyte formation was minimal despite repeated crossings suggesting that male fertility was impaired.

### Mp*gsnor1* mutations reduce GSNOR1 enzymatic activity

To assess the impact of Mp*GSNOR1* mutations on enzyme function, we quantified GSNOR activity in protein extracts from WT and mutant plants using a spectrophotometric assay that monitors GSNO-dependent NADH consumption at 340 nm. In this assay, GSNOR activity is reflected by the rate of decrease in NADH absorbance, which occurs as GSNO is reduced in an NADH-dependent reaction.

**Fig. 5.**
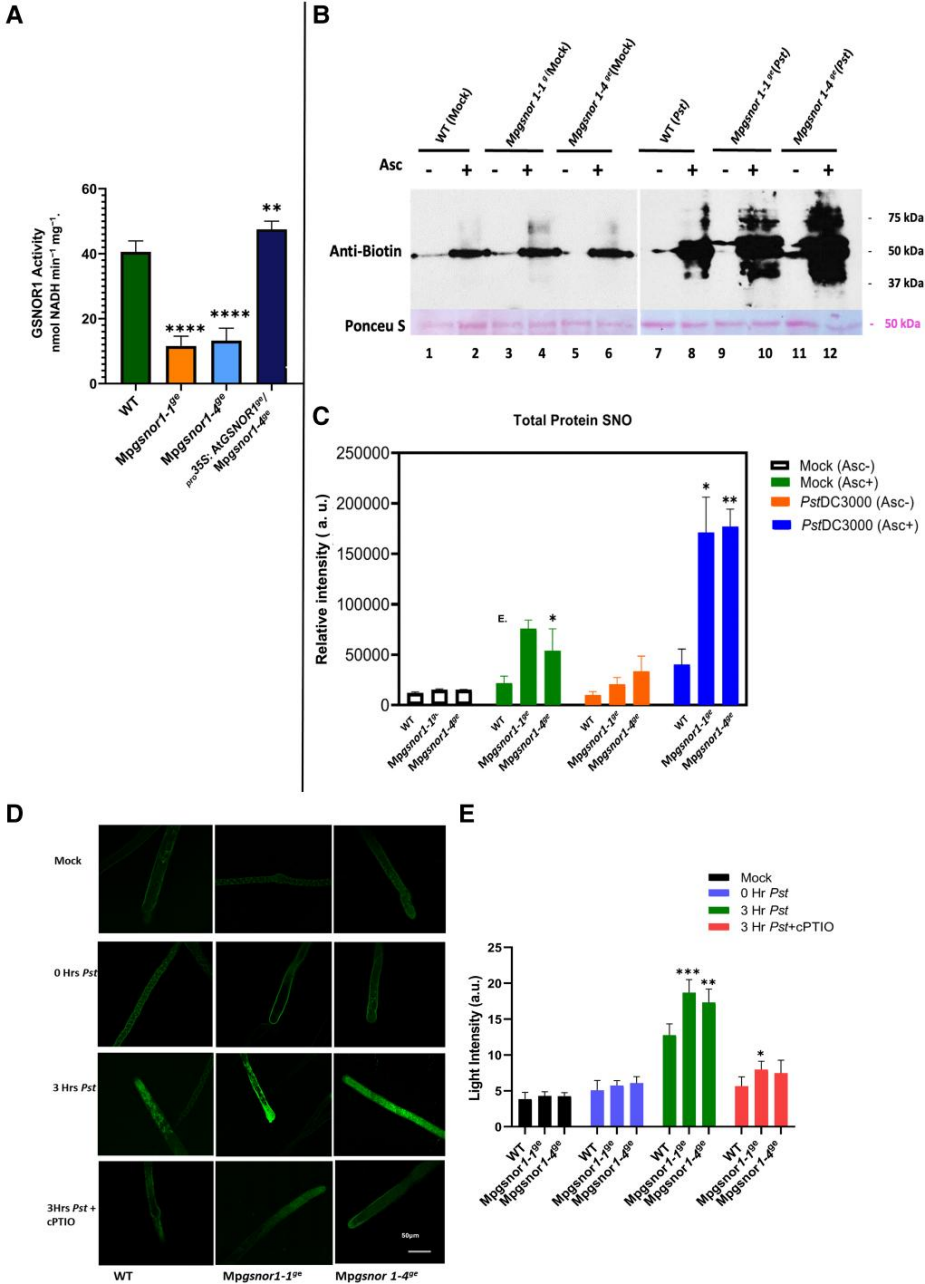
Comparative analysis of *S*-nitrosoglutathione reductase 1 (GSNOR1) enzymatic activity, total *S*-nitrosothiol (SNO) levels, and nitric oxide (NO) accumulation in response to pathogen infection. (A) GSNOR1 enzymatic activity measured as NADH oxidation rate per mg protein and expressed relative to wild-type (WT, Tak-1) (*n*=5). (B) Total SNO levels, measured by biotin-switch assay of protein extracts from WT and Mp*gsnor1* mutants after *Pseudomonas syringae* pv *tomato* (*Pst*) DC3000 or mock inoculation. Ascorbate (Asc) was included as a specificity control for the biotin-switch reaction. The immunoblot shown is representative of three independent biological experiments. (C) Quantification of total SNO levels. Signal intensities were quantified with ImageJ. Bar graph: white, mock/−Asc; green, mock/+Asc; orange, *Pst*/−Asc; blue, *Pst*/+Asc. (D) Infection with *Pst* DC3000 induces NO accumulation. Endogenous NO levels were examined in WT and Mp*gsnor1* mutants using diaminofluorescein-FM diacetate (DAF-FM DA) fluorescence staining. Rhizoids were subjected to *Pst* DC3000 infection, mock treatment (10 mM MgCl_2_), or treatment with the NO scavenger 2-(4-carboxyphenyl)-4,4,5,5-tetramethylimidazoline-1-oxyl-3-oxide (cPTIO) and stained with DAF-FM DA and imaged at the indicated time points. (E) Relative fluorescence intensity was quantified using ImageJ software. Data are presented as means ±SD from five biological replicates per genotype (*n*=5). The experiment was independently repeated three times with similar results. Error bars show SEM except (E) in which they are SD. Significance compared with WT is indicated (*P<0.05, **P<0.01, ***P<0.01, ****P<0.0001, unpaired Student’s *t*-test).

Both Mp*GSNOR1* mutant alleles exhibited a strong reduction in enzymatic activity compared with WT (Tak-1) plants (Fig. 5A). Specifically, GSNOR activity in Mp*gsnor1-1^ge^* and Mp*gsnor1-4^ge^* was reduced to approximately 29% and 33% of WT levels, respectively, indicating a substantial loss of function. To determine if Arabidopsis GSNOR1 can compensate for the loss of function in Mp*GSNOR1* mutants, we analysed the enzymatic activity in the *_pro_35S*:At*GSNOR1/*Mp*gsnor1-4^ge^* complementation line. The complementation line exhibited increased (120%) GSNOR1 activity relative to WT confirming that At*GSNOR1* effectively restores enzymatic function in Mp*GSNOR1* mutants (Fig. 5A).

These findings highlight the essential role of Mp*GSNOR1* in regulating nitrosative stress and demonstrate functional conservation between *Marchantia* and Arabidopsis GSNOR1 in maintaining redox homeostasis.

### Role of MpGSNOR1 in *S*-nitrosothiol homeostasis and immunity

To determine the role of MpGSNOR1 in immunity, we first characterized SNO levels in mutant and WT plants following infection by *Pst* DC3000. Plants were treated with a bacterial suspension of virulent *Pst* DC3000 or mock treated with a 10 mM MgCl_2_ solution as a control, then thallus tissue samples were collected 24 h post-inoculation, and total SNO levels were determined through the biotin switch assay (Jaffrey and Snyder, 2001). The results were striking: *Pst* DC3000-challenged plants, exhibited a significant increase in SNO levels compared with mock treated plants, but the increase was much greater in the mutants than in WT (Fig. 5B, C). This suggests that the absence of Mp*GSNOR1* compromises the regulation of *S*-nitrosylation, leading to a hyper-accumulation of SNOs. The enhanced SNO levels are consistent with *Pst* DC3000-induced NO production and subsequent SNO formation, a phenomenon previously reported in Arabidopsis (Feechan *et al*., 2005; Lee *et al*., 2008; Romero-Puertas *et al*., 2008; Tada *et al*., 2008; Hussain *et al*., 2019), and support a conserved role for GSNOR1 in regulating NO/SNO homeostasis during plant immune responses.

Following the increased SNO levels detected by the biotin-switch assay, we examined whether *Pst* DC3000 infection triggers a NO burst during basal immunity in *Marchantia*. WT (Tak-1) and MpGSNOR1 mutant plants (Mp*gsnor1-1^ge^* and Mp*gsnor1-4^ge^*) were inoculated with *Pst* DC3000, and endogenous NO levels were monitored using DAF-FM DA staining and confocal microscopy as described in the ‘Materials and methods’ (Fig. 5D, E). NO imaging was performed in rhizoids due to their translucent, root hair-like structure. Both WT and mutant plants showed pathogen-induced NO accumulation; however, NO levels were markedly higher in Mp*GSNOR1* mutants, with a pronounced increase at 3 h post-inoculation. Treatment with the NO scavenger cPTIO significantly reduced NO-associated fluorescence in all genotypes (Fig. 5D, E), confirming signal specificity. These results indicate that *Pst* DC3000 infection induces an early NO burst in *Marchantia*, which is enhanced in the absence of functional MpGSNOR1.

**Fig. 6.**
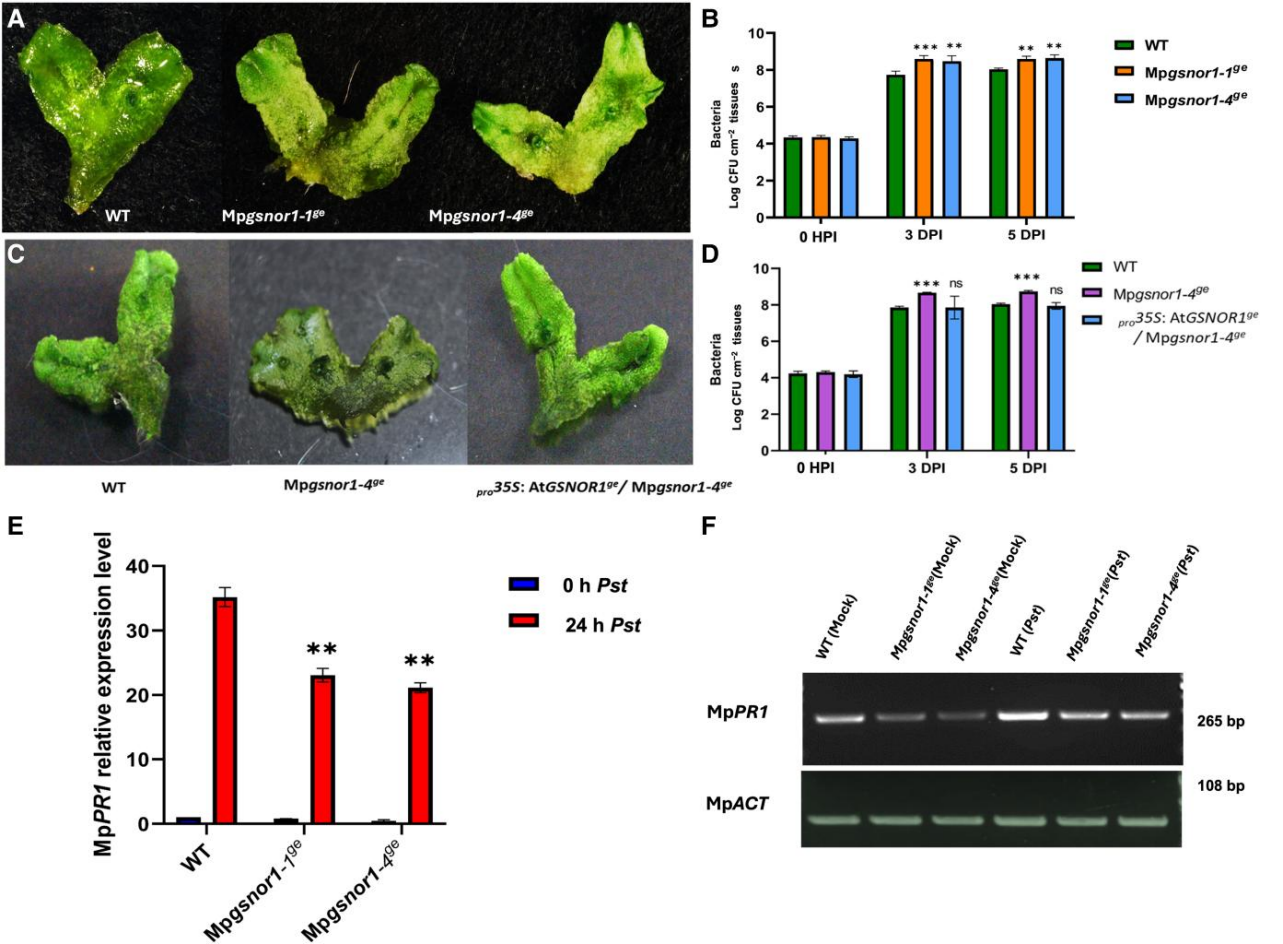
*Marchantia polymorpha S*-*nitrosoglutathione reductase 1* (Mp*GSNOR1*) contributes to resistance against *Pseudomonas syringae* pv. *tomato* (*Pst*) DC3000 and regulates defence gene expression. (A) Representative disease symptoms and bacterial growth (CFU cm^−2^) are shown for wild type (WT, Tak1) and mutant lines. (B) Quantitative analysis of bacterial proliferation in WT and Mp*gsnor1* mutant lines. Bacterial colonies of *Pst* DC3000 were quantified at different time points (HPI, hours post-inoculation; DPI, days post-inoculation). (C) Disease symptoms and bacterial growth, observed in both basal and apical thallus regions, are shown for WT, mutant, and the complementation line after inoculation with *Pst* DC3000. (D) Quantification of disease symptoms (*n*=3). (E) Quantitative real-time PCR (qRT-PCR) analysis of Mp*PR1* expression 24 h after *Pst* DC3000 challenge. Expression levels are presented as fold change relative to mock-treated controls. Error bars represent SEM from three independent biological replicates. Asterisks indicate statistically significant differences compared with 0 h controls (*P*<0.05). (F) Semi-quantitative RT-PCR (sqRT-PCR) analysis by gel electrophoresis showing Mp*PR1* expression 24 h after *Pst* DC3000 inoculation. Mp*ACT* was used as a housekeeping gene, and expression levels were compared with 0 h controls. In all plots, error bars represent SEM (*n*=5). Significant differences between Mp*GSNOR1* mutants and WT plants are indicated: ns, not significant; *P<0.05, **P<0.01, ***P<0.01, ****P<0.0001, based on an unpaired Student’s *t*-test.

We next investigated the role of MpGSNOR1 in basal disease resistance, by assessing the susceptibility of Mp*gsnor1-1^ge^* and Mp*gsnor1-4^ge^* mutants to *Pst* DC3000. Three-week-old thalli from WT and mutant plants were inoculated with *Pst* DC3000 at a concentration of 10^8^ cfu ml^−1^. Bacterial proliferation was quantified at 0, 3 and 5 d post-inoculation following the protocol described by Gimenez-Ibáñez *et al*. (2019). Both mutants exhibited significantly increased bacterial growth compared with WT plants (Fig. 6A, B). At 3 d and 5 d post-inoculation, bacterial densities in the mutants reached 10^8^–10^9^ cfu cm^−2^, whereas WT plants restricted growth to approximately 10^7^ cfu cm^−2^. Symptoms were most pronounced at the basal regions of the thalli, although bacterial colonization was also observed at the apical notches. A progressive increase in susceptibility was evident over time, with more severe symptoms by day 5. To determine whether Arabidopsis *GSNOR1* could restore *Pst* DC3000 resistance, the *_pro_35S*:At*GSNOR1*^ge^/Mp*gsnor1-4^ge^* complementation line was analysed. This line showed enhanced resistance, correlating with reduced bacterial loads and attenuated disease symptoms (Fig. 6C, D). These results suggest that Mp*GSNOR1* is essential for basal resistance to *Pst* Dc3000. The functional complementation by At*GSNOR1* confirms its conserved role in immune responses. Our findings suggest that GSNOR1-mediated redox homeostasis is a pivotal factor in plant defence mechanisms across species.

### Mpgsnor1 compromises salicylic acid-regulated gene expression

SA is a key defence hormone, essential for plant immunity against *Pst* DC3000. While primarily studied in angiosperms, SA signalling is evolutionarily conserved in bryophytes, including *Marchantia* (Gimenez-Ibáñez *et al*., 2019). To investigate the role of Mp*GSNOR1* in SA-mediated defence, we analysed the expression of SA-related genes in WT and Mp*GSNOR1* mutant plants following *Pst* DC3000 infection. qRT-PCR revealed significantly reduced expression of *Pathogenesis-Related 1* (*PR1*), a marker of SA signalling, in Mp*gsnor1-1^ge^* and Mp*gsnor1-4^ge^* mutants compared with WT plants 24 h post-infection, indicating impaired SA-dependent defence activation (Fig. 6E). Similar results were observed with sqRT-PCR by gel electrophoresis (Fig. 6F).

While Mp*PR1* expression responds to pathogen challenge, its precise relationship to SA signalling in *Marchantia* remains unclear. Nonetheless, our findings indicate that MpGSNOR1 plays an important role in regulating defence-related transcriptional responses during basal resistance to *Pst* DC3000.

## Discussion

### GSNOR1 as an evolutionarily conserved regulator of NO signalling

Our study showed that GSNOR1 has a conserved and indispensable role in NO homeostasis, development, and immunity in *Marchantia*. Our phylogenetic analysis indicates that GSNOR is conserved in all lineages of the *Archaeplastida*, illustrating the importance of this enzyme for core biological processes (Fig. 1). GSNOR was present in the common ancestor of land plants and retained in *Marchantia*, where it is encoded by a single gene, Mp*GSNOR1*, the orthologue of Arabidopsis At*GSNOR1*. The two proteins share extensive sequence and structural similarity throughout their lengths, highlighting the evolutionary constraint on this enzyme and its non-redundant function in plant biology (Fig. 2) (Lynch and Conery, 2000; Xu *et al*., 2013).

To further understand how this conservation relates to GSNOR1 regulation, we compared the amino acid sequences of MpGSNOR1 and AtGSNOR1 and examined key residues known to modulate enzyme activity through PTMs. This analysis (Supplementary Fig. S1) revealed that MpGSNOR1 conserves all major catalytic, structural, cofactor-binding, and substrate-binding residues previously characterized in AtGSNOR1 (Kubienová *et al*., 2013; Xu *et al*., 2013). Importantly, several solvent-accessible cysteine residues, targets of redox-based post-translational modifications such as *S*-nitrosylation and *S*-glutathionylation in AtGSNOR1 (Leterrier *et al*., 2011), are also conserved in MpGSNOR1. This conservation suggests that redox-dependent regulatory mechanisms controlling GSNOR1 activity are evolutionarily ancient and were likely already present in early-diverging land plants.

In addition, the conservation of the overall domain architecture between MpGSNOR1 and AtGSNOR1 (Supplementary Fig. S2) provides further evidence for a strong evolutionary constraint on this enzyme. The retention of the canonical MDR/GSNOR domain in *Marchantia* indicates that the structural basis for GSNOR catalytic function predates the divergence of bryophytes and angiosperms. Together with the preservation of PTM-sensitive residues, this supports the idea that the coupling of GSNOR activity to redox homeostasis is a conserved regulatory strategy within plant NO signalling pathways.

### Functional impact and compensatory pathways in Mp*GSNOR1* mutants

We found that although GSNOR enzyme activity is strongly reduced in Mp*gsnor1-1^ge^* and Mp*gsnor1-4^ge^* mutants to 29–33% of WT levels, it is not completely eliminated. This residual activity is similar to what was observed in Arabidopsis At*GSNOR1* mutants (Fig. 5) (Feechan *et al*., 2005) and suggests that alternative pathways can partially compensate for the loss of GSNOR activity. Recent biochemical studies highlight that aldo–keto reductases (AKRs) may play a role in GSNO metabolism during stress (Stomberski *et al*., 2019; Treffon and Vierling, 2022), although there is no genetic evidence to support functional compensation of GSNOR by aldo–keto reductases *in planta*. In Arabidopsis, AKR4C8 and similar members of its clade show enzymatic properties comparable to the functions of mammalian AKR1A1 and can reduce GSNO albeit with less catalytic efficiency and specificity than GSNOR (Treffon *et al*., 2021; Treffon and Vierling, 2022). Therefore, one possibility is that AKRs or other enzymes work in part to replace GSNOR activity in the control of GSNO and nitrosative stress in MpG*SNOR1* mutants. These alternative pathways need more biochemical and genetic investigation.

### Nitric oxide homeostasis and the many functions of GSNOR1 in *Marchantia* development

NO is a central signalling molecule in plants, with *S*-nitrosylation modulating key physiological processes and stress responses (Melotto *et al*., 2006; Besson-Bard *et al*., 2008a, b; Allain *et al*., 2011; Gaupels *et al*., 2011). In our examination of CRISPR/Cas9-induced Mp*GSNOR1* mutants, we found that there is a significant alteration of the vegetative and reproductive development (Fig. 4). These included smaller plant size, smaller thalli, and different thallus shapes (Fig. 3). The mutants had shorter and fewer rhizoids, along with significant delays and reductions in sexual structures such as antheridia and sporophytes (Fig. 4). These alterations were similar to those observed in the developmental and reproductive structures in Arabidopsis *GSNOR1* mutants (Kwon *et al*., 2012). These findings point to the critical role of GSNOR1 in reproductive development, likely through NO-regulated pathways that control organ formation and light-responsive transitions (Naramoto *et al*., 2019).

These phenotypic abnormalities may reflect perturbations in hormonal pathways. Mp*GSNOR1* mutations likely impact auxin, ethylene, and cytokinin pathways that are essential for *Marchantia* development (Kato *et al*., 2017; Aki *et al*., 2019; Katayose *et al*., 2021; Pande *et al*., 2022). For instance, NO-mediated *S*-nitrosylation has been shown to alter the activity of auxin receptors (TIR1/AFBs) and response factors in Arabidopsis (Fernández-Marcos *et al*., 2011; Terrile *et al*., 2012; Iglesias *et al*., 2018; Pande *et al*., 2022), and these auxin signalling pathway components are conserved in *Marchantia* (Eklund *et al*., 2015; Flores-Sandoval *et al*., 2015; Kato *et al*., 2017; Suzuki *et al*., 2023). Changes in NO balance in Mp*GSNOR1* mutants may therefore disrupt auxin signalling, which affects growth. Similarly, *S*-nitrosylation impacts the biosynthesis and action of ethylene. Enzymes like ACC synthase and ACC oxidase, along with their regulators, are sensitive to NO status (Pattyn *et al*., 2021; Li *et al*., 2022). These pathways might be further explored using ethylene-specific inhibitors of biosynthesis and perception (Lindermayr *et al*., 2006; Depaepe and Van Der Straeten, 2016; Li *et al*., 2022; Qi *et al*., 2022).

Cytokinin signalling is crucial for gemma cup formation and is regulated by genes like Mp*RRB* and Mp*GCAM1* (Aki *et al*., 2019, 2022). Imbalances between auxin and cytokinin could explain some developmental defects in Mp*gsnor1* mutants. Experimentally, NO has been found to change cytokinin signalling in a directed manner (Feng *et al*., 2013; Pande *et al*., 2022). In particular, *S*-nitrosylation of key signalling molecules in Arabidopsis, including HISTIDINE PHOSPHOTRANSFER PROTEIN 1 (AHP1), cleaves the phosphorelay cascade that is central to cytokinin signalling (Feng *et al*., 2013; Pande *et al*., 2022). Cytokinin signalling components including AHP1 are conserved in *Marchantia*, so it is possible that similar regulation by SNO contributes to the effects seen on gemma cup formation and development.

The traits seen in Mp*GSNOR1* mutants may indicate broader changes in hormonal signalling networks and transcriptional regulation. These changes could affect morphogenesis, rhizoid development, and reproduction. While more hormonal and signalling studies are necessary, these findings suggest that GSNOR1 has a complex role in *Marchantia*. They also help us understand the shared mechanisms that drive plant growth, development, and adaptation.

### Evolutionary conservation of Mp*GSNOR1* in immune regulation

GSNOR1 plays a key role in maintaining the balance of specific cellular molecules by controlling global *S*-nitrosylation (Feechan *et al*., 2005; Kwon *et al*., 2012). In Arabidopsis, the loss of At*GSNOR1* results in impaired GSNO breakdown, leading to higher levels of *S*-nitrosylated proteins and GSNO (Feechan *et al*., 2005; Tada *et al*., 2008). *GSNOR1* itself can be modified by *S*-nitrosylation, particularly at the Cys-10 residue, and this change has been shown to reduce its enzymatic activity in both plants and other organisms (Lee *et al*., 2008; Kubienová *et al*., 2013; Zhan *et al*., 2018). These redox modifications of GSNOR1 highlight the complex nature of NO signalling in plant cells.

Studies using reverse genetics have shown that GSNOR1 plays a central role in controlling *S*-nitrosylation and the expression of SA-dependent genes, which in turn shape the immune responses of vascular plants (Feechan *et al*., 2005; Xia *et al*., 2014). However, the role of GSNOR1 in non-vascular plants remained largely unknown. By investigating *Marchantia* (Bowman *et al*., 2017, 2022; Alcaraz *et al*., 2018), our study addresses this gap and provides new insights into the evolutionary origins of NO-based immunity.

Our findings demonstrate that Mp*GSNOR1* mutants accumulate higher levels of SNO proteins, indicating that the absence of *GSNOR1* disrupts NO-dependent signalling networks (Fig. 5). Consistent with findings in angiosperms, these results support the hypothesis that SNOs act as key mediators of NO bioactivity during pathogen challenge and contribute to plant disease resistance through coordinated roles in redox regulation and defence signalling (Feechan *et al*., 2005; Lee *et al*., 2008; Tada *et al*., 2008; Hussain *et al*., 2019).

Phytohormones such as SA and jasmonate are central to immune regulation in both *Marchantia* and angiosperms (Dempsey *et al*., 2011; Boatwright and Pajerowska-Mukhtar, 2013; Gimenez-Ibáñez *et al*., 2019; Monte, 2023). Exposure to pathogens results in up-regulation of SA marker genes in *Marchantia*, indicative of an ancient role for SA-mediated immunity (Gimenez-Ibáñez *et al*., 2019). Consistent with this, Mp*GSNOR1* mutants show reduced SA-dependent gene expression, including lower Mp*PR1* levels, highlighting a conserved role for GSNOR1 in modulating SA signalling (Fig. 6) (Feechan *et al*., 2005). Interestingly, the perception of SA in *Marchantia* appears to differ from that in Arabidopsis, at least as regards immunity. In Arabidopsis, SA signalling is mediated by the receptor NPR1 together with its cofactor TGA1 (Després *et al*., 2003; Shearer *et al*., 2012), so that SA-induced transcriptional reprogramming is lost in the *npr1* mutants. *Marchantia* contains single copies of *NPR1* and *TGA1*, suggesting that NPR-dependent SA signalling was present in the most recent common ancestor of land plants (Jia *et al*., 2023). Functional studies show that although MpNPR1 can bind SA, mutants are hypersensitive to SA and the transcriptional reprogramming that occurs in response to SA is largely intact in Mp*npr1* mutants, implying the existence of an alternative SA sensing pathway in *Marchantia* (Jeon *et al*, 2024). In addition, although *S*-nitrosylation of *NPR1* at Cys-156 by GSNO promotes its oligomerization and stabilizes protein homeostasis in response to SA in Arabidopsis (Tada *et al*., 2008) comparative sequence analysis of 194 NPR proteins found that Cys156 is conserved only in *Brassicaceae* so it is not clear whether MpNPR1 is regulated by *S*-nitrosylation of cysteine residues (Jeon *et al*., 2024). It will be interesting to determine what the alternative SA signalling pathways are and whether their components are SNO regulated.

The bacterial pathogen *Pst* DC3000 has been identified as a virulent agent in *Marchantia* (Gimenez-Ibáñez *et al*., 2019). Our results show that Mp*GSNOR1* mutants are more susceptible to *Pst* DC3000, whereas complementation with At*GSNOR1* restores resistance (Fig. 6). This functional conservation underscores the broad importance of *GSNOR1* across land plants, as Arabidopsis *GSNOR1* mutants similarly exhibit increased pathogen susceptibility (Feechan *et al*., 2005). Moreover, our findings complement recent studies demonstrating both functional compatibility (Grenz *et al*., 2025) and ecological relevance (Robinson *et al*., 2025) of *Pseudomonas–Marchantia* interactions, further supporting the use of this system to investigate conserved immune and redox-regulatory mechanisms.

The cross-species complementation of Mp*GSNOR1* mutants with At*GSNOR1*, resulting in restored pathogen resistance, provides direct evidence for the evolutionary conservation of GSNOR1-mediated immune regulation.

### Conclusions and future directions

**Fig. 7.**
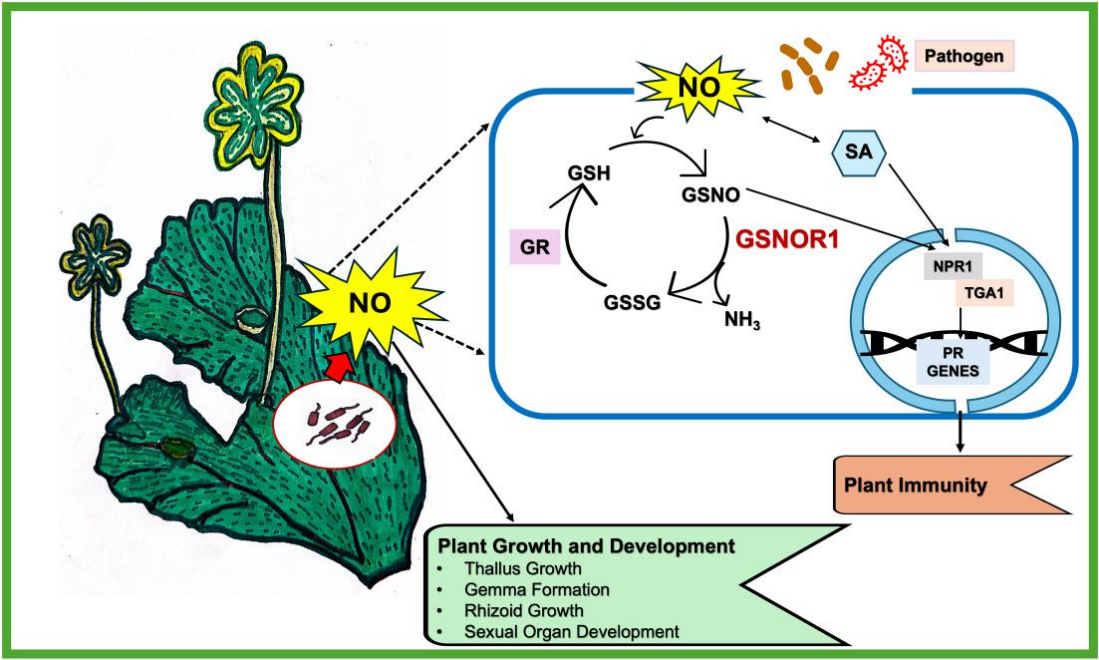
Schematic overview of *S*-nitrosoglutathione reductase 1 (GSNOR1) function in *Marchantia*. This diagram outlines the role of GSNOR1 in plant immunity and development in the non-vascular plant *Marchantia*. It shows how GSNOR1 indirectly regulates salicylic acid (SA)-mediated defence by controlling NO levels through GSNO degradation. Upon pathogen detection, SA synthesis is triggered, and GSNOR1 modulates this signalling to activate defence genes. The figure also highlights the crosstalk between SA and nitric oxide (NO) pathways mediated by GSNOR1. Beyond immunity, GSNOR1 influences developmental processes, including thallus growth, gemma formation, rhizoid development, and reproductive organ formation by maintaining NO balance, which is vital for environmental adaptation. GR, glutathione reductase; GSH, reduced glutathione; GSNO, *S*-nitrosoglutathione; GSSG, oxidized glutathione; PR, pathogenesis-related.

Our study establishes GSNOR1 as a central, evolutionarily conserved regulator of NO homeostasis, hormone signalling, development, and immunity in *Marchantia* (Fig. 7). While the fundamental roles of GSNOR1 are shared across land plants, species-specific adaptations in hormonal and immune responses persist. Future work should aim to biochemically and genetically dissect the compensatory roles of AKRs and elucidate the molecular interplay between GSNOR1 and NPR1 or alternative SA receptors in non-vascular plants, paving the way for targeted improvements in crop resilience and disease resistance.

## Supplementary Material

erag171_Supplementary_Data

## Data Availability

All data generated or analysed in this study are available within the published article and its supplementary materials.

## Supplementary data

The following supplementary data are available at *JXB* online.

Fig. S1. Sequence alignments of GSNOR1 proteins showing conserved catalytic structure and binding residues.

Fig. S2. Comparative conserved domain architecture of AtGSNOR1 and MpGSNOR1 proteins.

Fig. S3. Details of MpGSNOR1 gene edited alleles.

Fig. S4. Rhizoid morphology in wild type and Mp*gsnor1* mutants.

Fig. S5. Gemma cup development in wild type, Mp*gsnor1* mutants, and complementation line.

Table S1. List of primers used.

## Abbreviations

Cys: cysteine
GSNO: *S*-nitrosoglutathione
GSNOR: *S*-nitrosoglutathione reductase
NO: nitric oxide
qRT-PCR: quantitative real-time PCR
SA: salicylic acid
SNO: *S*-nitrosothiol
Tak-1: Takaragaike-1
WT: wild type
