# Supplementary material for: A key role for S-nitrosylation in immune regulation and development in the liverwort Marchantia polymorpha
Source: J Exp Bot. 2026 Apr 15;77(14):4561–78. doi: 10.1093/jxb/erag171 (PMC13415969; doi:10.1093/jxb/erag171)
Supplement: erag171_Supplementary_Data [file erag171_supplementary_data.zip › jexbot317857-file001.pdf]

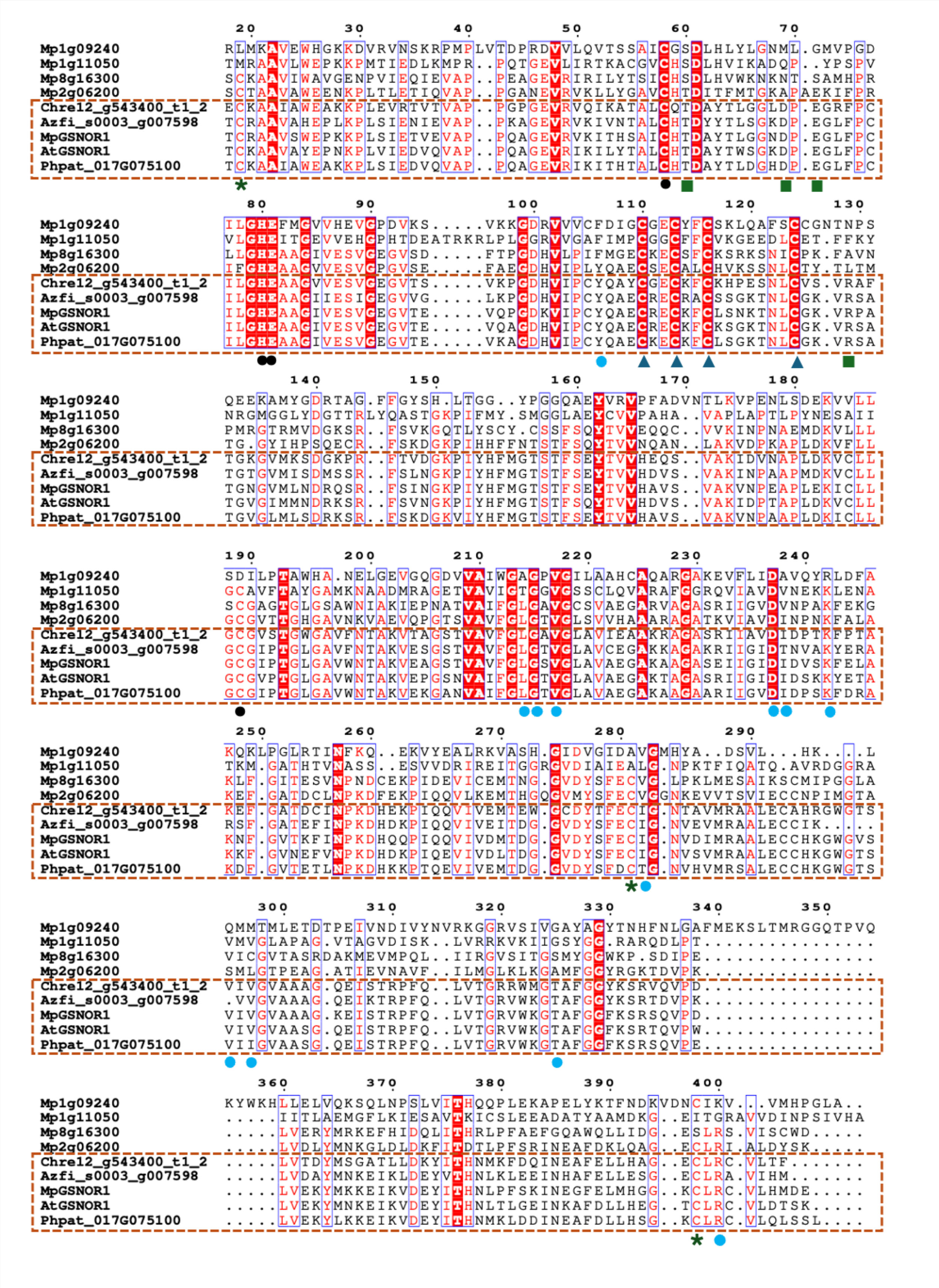

Figure S1: Conservation of Catalytic, Structural, and Binding Residues in Plant GSNORs.

Multiple sequence alignment of GSNOR proteins from Marchantia, moss, and green algae. Amino acid sequences were aligned using Clustal Omega (Sievers et al., 2011) and visualised with ESPript 3.0 (Robert & Gouet, 2014). Species abbreviations: At – *Arabidopsis thaliana*; Azfi – *Azolla filiculoides*; Phpa – *Physcomitrella patens*; Mp – *Marchantia polymorpha*; Chre – *Chlamydomonas reinhardtii*. Residue functional annotations are based on structural and biochemical studies of *Arabidopsis thaliana* GSNOR (e.g., Kubienová et al., 2013; Leterrier et al., 2011). Black filled circles (●) indicate residues coordinating the catalytic zinc atom; black filled triangles (▲) mark residues coordinating the structural zinc atom; blue filled circles (🔵) denote residues interacting with the NAD(H) cofactor; green squares (🟩) indicate substrate-binding residues (GSNO/HMGSH). Asterisks (*) mark solvent-accessible cysteine residues potentially targeted for redox-based post-translational modifications. Orthologs from different species are enclosed within dotted boxes to highlight sequence conservation.

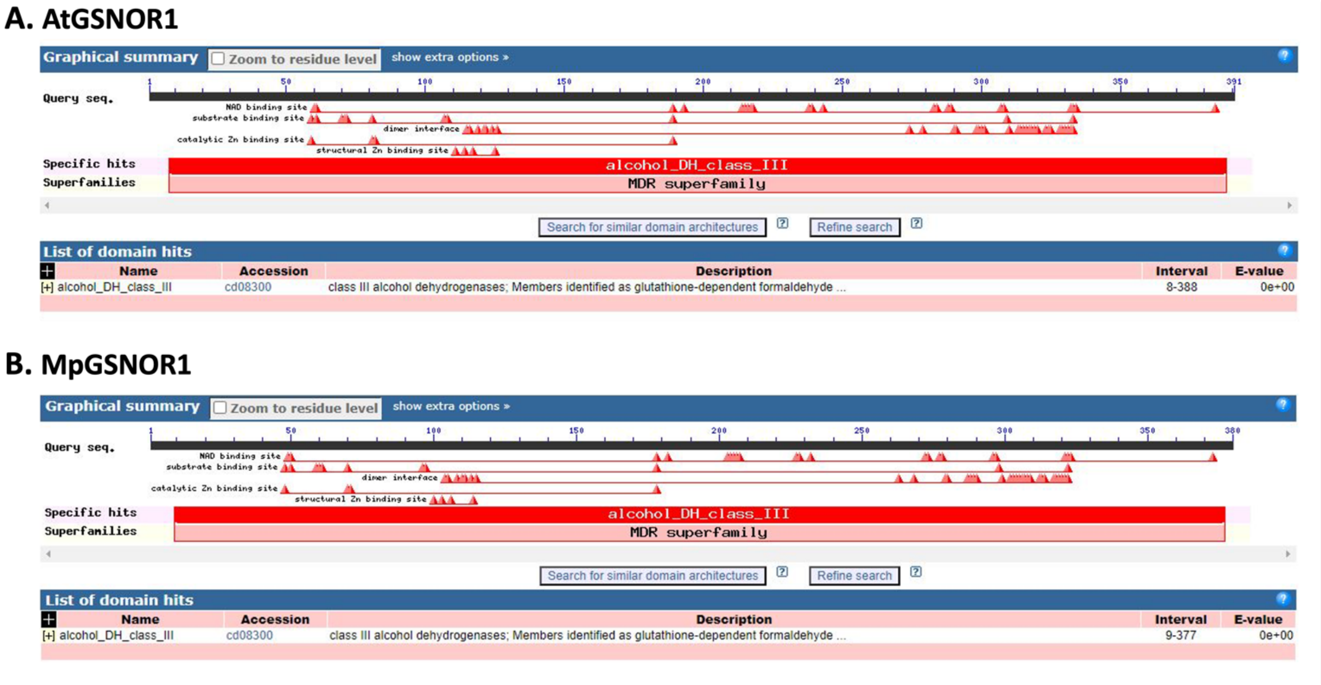

Figure S2. Comparative conserved domain architecture of AtGSNOR1 and MpGSNOR1 proteins.

This figure presents a comparative analysis of the conserved domain structures of GSNOR1 proteins from A. AtGSNOR1 (*Arabidopsis thaliana*) and B. MpGSNOR1 (*Marchantia polymorpha*) using the Conserved Domain Database (CDD) of NCBI (Domain architecture ID: 10169723 for AtGSNOR1). In both proteins, conserved domains are depicted as red ribbons aligned along their respective amino acid sequences, with interruptions indicating non-conserved regions or gaps in the domains.

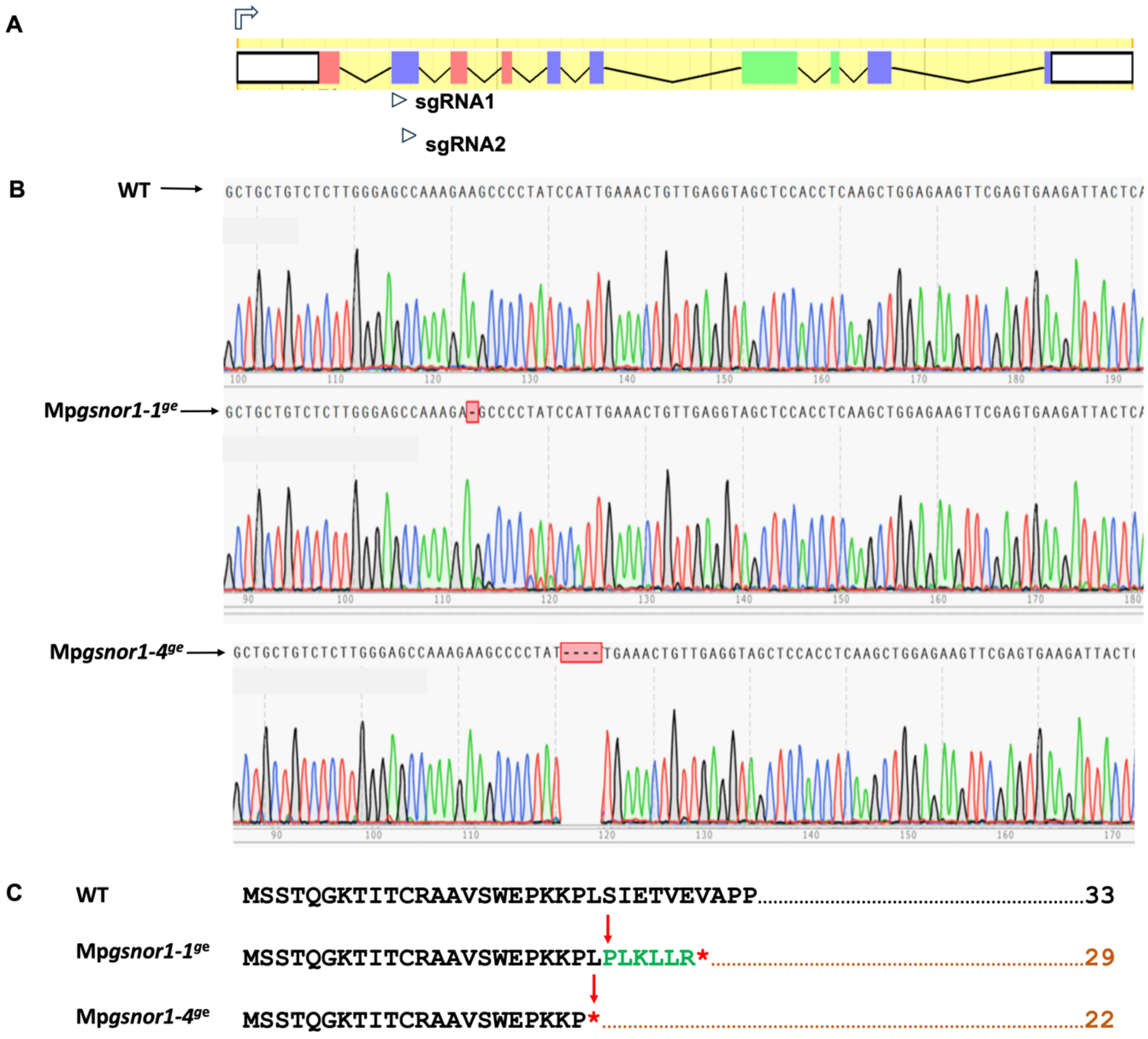

Figure S3. Sanger sequencing chromatograms showing CRISPR/Cas9-generated mutations in Exon 2 of the Mp*GSNOR1* gene and the resulting frameshift-mediated disruption of the predicted protein sequence.

(A) Schematic representation of the Mp*GSNOR1* gene. White boxes represent untranslated regions (UTRs), while coloured boxes indicate protein-coding exons (CDS). Black connecting lines correspond to introns. The positions of the two single-guide RNAs (sgRNA1 and sgRNA2) used for genome editing are shown.
(B) Chromatograms displaying the Exon 2 sequence for the wild type (WT), a single-nucleotide deletion (Mp*gsnor1-1^{ge}*) and a 4-nucleotide deletion (Mp*gsnor1-4^{ge}*) mutant. These mutations result in frameshifts that are predicted to disrupt the function of the MpGSNOR1 protein.
(C) Comparative analysis of predicted amino-acid sequences of the WT MpGSNOR1 protein and the two mutant alleles (Mp*gsnor1-1^{ge}* and Mp*gsnor1-4^{ge}*), obtained by translating coding sequences in the 5′→3′ direction. CRISPR/Cas9-induced deletions and corresponding changes in the downstream amino acid sequence are shown; stop codons are indicated by (*), and the corresponding residue numbers at the truncation sites are shown. Red arrows indicate the position at which mutant alleles diverge from the WT protein sequence.

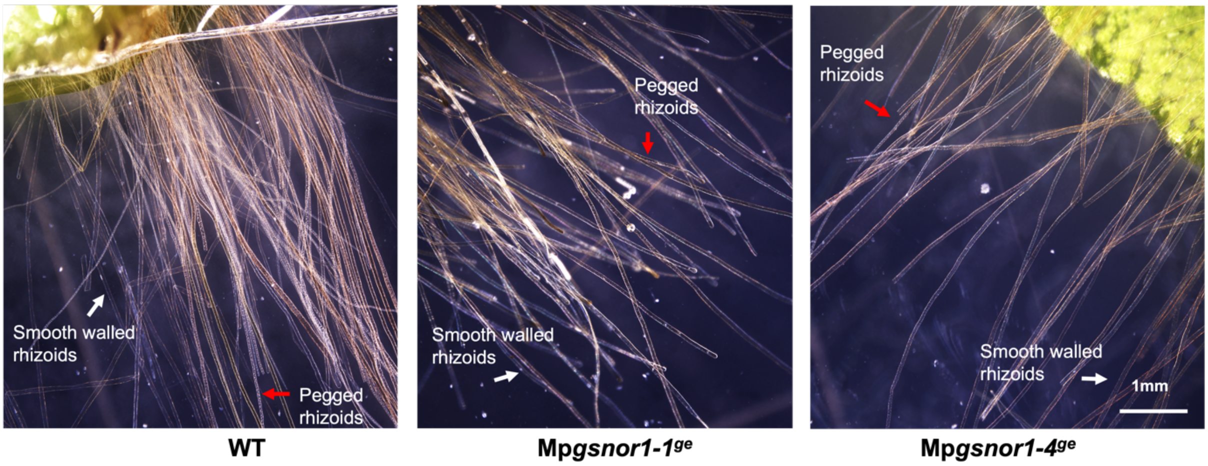

Figure S4. Rhizoid morphology in wild-type and Mp*GSNOR1* loss-of-function mutant plants.

Representative microscopic images showing rhizoids of wild-type, Mp*gsnor1-1*^{ge} and Mp*gsnor1-4*^{ge} mutant plants. Both smooth and pegged rhizoids are observed in all genotypes. Images were acquired using a Nikon SMZ1500 microscope. Scale bar = 1 mm.

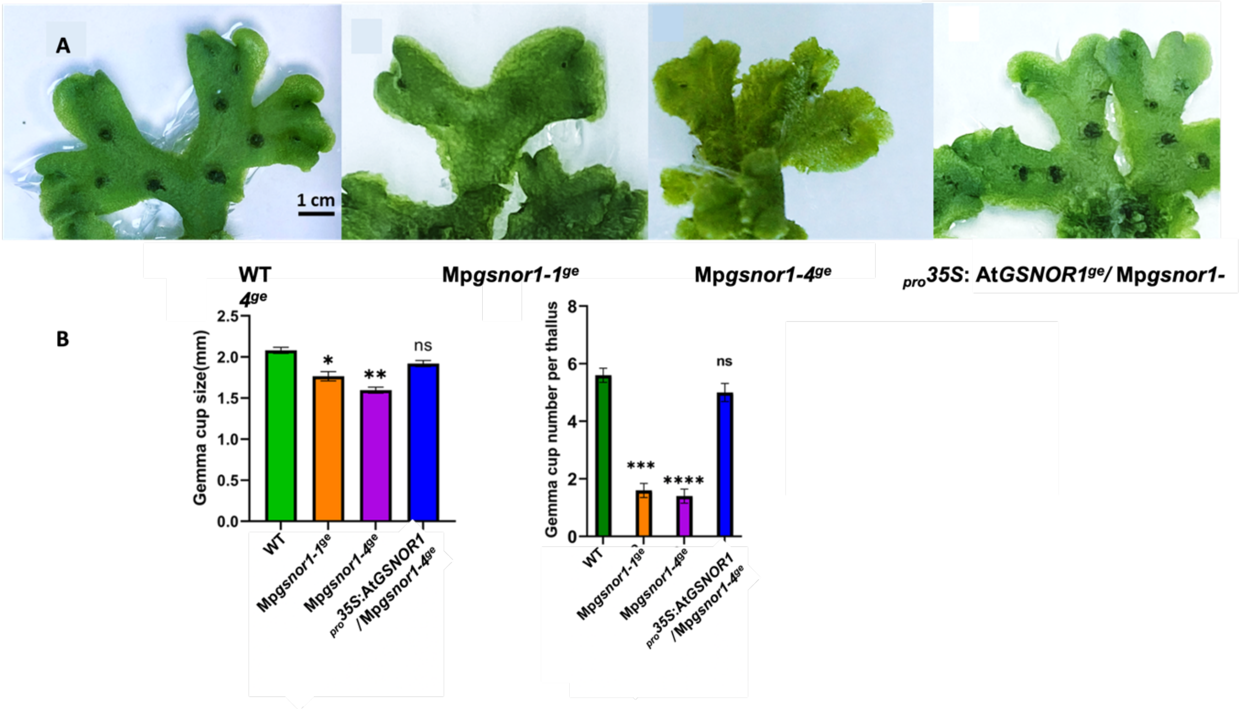

Figure S5: Comparative assessment of gemma cup development and gemma size in Marchantia wild-type, mutant, and complementation lines.

The figure compares gemma production and size in the (A) Wild-type, (B) Mp*gsnor1-1*^{ge} and (C) Mp*gsnor1-4*^{ge} mutants, and (D) the complementation line $_{pro}$*35S*: At*GSNOR1*^{ge}/Mp*gsnor1-4*^{ge}. Bars depict mean values ± SEM (n = 5), with statistical significance relative to wild-type indicated (ns = non-significant, *$p < 0.05$, **$p < 0.01$, ***$p < 0.001$,****$p < 0.0001$; unpaired Student's t-test). Scale bars = 1 cm.
