# Supplementary material for: A key role for S-nitrosylation in immune regulation and development in the liverwort Marchantia polymorpha
Source: J Exp Bot. 2026 Apr 15;77(14):4561–78. doi: 10.1093/jxb/erag171 (PMC13415969; doi:10.1093/jxb/erag171)
Supplement: erag171_Supplementary_Data [file erag171_supplementary_data.zip › jexbot317857-file004.pdf]

**Table S1- List of primers used for this study**

| Purpose | Primer Name | Gene ID | Primer Sequence (5’ to 3’) |
|---|---|---|---|
| guide RNAs to target Mp*GSNOR1* | MpGNR_sgRNA1 F | Mp1g16170 | CTCG TCAATGGATAGGGGCTTCTT |
| guide RNAs to target Mp*GSNOR1* | MpGNR_sgRNA1 R | Mp1g16170 | AAAC AAGAAGCCCCTATCCATTGA |
| guide RNAs to target Mp*GSNOR1* | MpGNR_sgRNA2 F | Mp1g16170 | CTCG TCAACAGTTTCAATGGATAG |
| guide RNAs to target Mp*GSNOR1* | MpGNR_sgRNA2 R | Mp1g16170 | AAAC CTATCCATTGAAACTGTTGA |
| screening transgenic generations | MpGNR0 F | Mp1g16170 | ATGAGTCAAGCGAAATTGTCGTAGT |
| screening transgenic generations | MpGNR0 R | Mp1g16170 | GAATGCAAGGAAAGAGACCTTCAGG |
| complementation construct | attB1-At*GSNOR1*–F | AT5G43940 | GGGGACAAGTTTGTACAAAAAAGCAGGC TTAATGGCGACTCAAGGTCAGG |
| complementation construct | attB1-At*GSNOR1*-R | AT5G43940 | GGGGACCACTTTGTACAAGAAAGCTGGG TTTCATTTGCTGGTATCGAGGACA |
| For screening complementation construct | At*GSNOR1*-F | AT5G43940 | ATGGCGACTCAAGGTCAGGT |

| | | | |
|---|---|---|---|
| For screening complementation construct | At*GSNOR1*- R | AT5G43940 | TCATTTGCTGGTATCGAGGA |
| for RT-PCR | Mp*PR1* F | **Mp3g14110.1** | TCGTAAGGCTGTCAAAGTCC |
| for RT-PCR | Mp*PR1* R | **Mp3g14110.1** | CAACCTGAGTGTAAATCCGAC |
| for RT-PCR | Mp*ACT* F | **Mp6g11010.1** | AGGCATCTGGTATCCACGAG |
| for RT-PCR | Mp*ACT* R | **Mp6g11010.1** | ACATGGTCGTTCCTCCAGAC |
